# Multi-trait association study identifies loci associated with tolerance of low phosphorus in *Oryza sativa* and its wild relatives

**DOI:** 10.1038/s41598-022-07781-5

**Published:** 2022-03-08

**Authors:** Annamalai Anandan, Ranjitkumar Nagireddy, Selvaraj Sabarinathan, Bishal Binaya Bhatta, Anumalla Mahender, Murugapandiyan Vinothkumar, Chidambaranathan Parameswaran, Periyasamy Panneerselvam, Hatanath Subudhi, Jitendriya Meher, Lotan Kumar Bose, Jauhar Ali

**Affiliations:** 1 Crop Improvement Division, ICAR-National Rice Research Institute (NRRI), Cuttack, Odisha 753006 India; 2 Department of Plant Physiology, Orissa University of Agriculture and Technology, Bhubaneswar, Odisha 751003 India; 3 Rice Breeding Innovation Platform, International Rice Research Institute (IRRI), 4031 Los Baños, Laguna Philippines; 4 Crop Production Division, ICAR-National Rice Research Institute (NRRI), Cuttack, Odisha 753006 India

**Keywords:** Genetics, Plant sciences

## Abstract

We studied variation in adaptive traits and genetic association to understand the low P responses, including the symbiotic association of arbuscular mycorrhizal (AM) fungal colonization in *Oryza* species (*O. sativa*, *O. nivara,* and *O. rufipogon*). In the present experiment, we performed the phenotypic variability of the morphometric and geometric traits for P deficiency tolerance and conducted the association studies in GLM and MLM methods. A positive association between the geometric trait of the top-view area and root traits suggested the possibility of exploring a non-destructive approach in screening genotypes under low P. The AMOVA revealed a higher proportion of variation among the individuals as they belonged to different species of *Oryza* and the NM value was 2.0, indicating possible gene flow between populations. A sub-cluster with superior-performing accessions had a higher proportion of landraces (42.85%), and *O. rufipogon* (33.3%) was differentiated by four *Pup1*-specific markers. Association mapping identified seven notable markers (RM259, RM297, RM30, RM6966, RM242, RM184, and PAP1) and six potential genotypes (IC459373, Chakhao Aumbi, AC100219, AC100062, Sekri, and Kumbhi Phou), which will be helpful in the marker-assisted breeding to improve rice for P-deprived condition. In addition, total root surface area becomes a single major trait that helps in P uptake under deficit P up to 33% than mycorrhizal colonization. Further, the phenotypic analysis of the morphometric and geometric trait variations and their interactions provides excellent potential for selecting donors for improving P-use efficiency. The identified potential candidate genes and markers offered new insights into our understanding of the molecular and physiological mechanisms driving PUE and improving grain yield under low-P conditions.

## Introduction

Phosphorus (P) is significant in that it affects the growth and productivity of plants. A high amount of P is usually present in the soil, but availability is limited as P usually exists in the organic form, carbonate (alkaline) or oxide (acidic)^1,2^. Low P availability is the major constraint in agriculture^3,4^, with 20–30% of the applied P readily available to plants and the remaining converted to unavailable forms, thus creating water eutrophication^5^. As rice is the major P utilizer, enhancing P-use efficiency (PUE) is highly required for sustainable production. About 20 million hectares of world rice cultivation area are under P deficiency^6^, while 61.02% of Indian soil is low in P^7^. Global commercial phosphate reserves are estimated to become depleted in 300 to 400 years^8^. Around 90% of the P-based fertilizer and raw materials are imported, and the 10% indigenous rock deposits hardly satisfy India's domestic market^9^. After the introduction of high-yielding varieties (HYVs), the consumption of phosphate fertilizer gradually rose to 6.86 million metric tons in 2017–18 from less than 1 million tons (0.132 million MT) during 1965–66 (https://www.faidelhi.org/statistics/statistical-database). This suggests that there is a need to develop varieties that use P more efficiently and effectively to decrease the phosphate fertilizers needed for sustainable rice production^10,11^.

Phosphorus is involved in plant growth and is a critical component of nucleic acid, membrane phospholipids, ATP, and NADPH. Upon P starvation, remodeling of cellular processes and high turnover of phospholipids into galactolipids and sulfolipids have been observed^12^. In addition, environmental factors were also reported to have a substantial effect on P-starved plants. Further, arrest in primary root growth occurs by affecting the cell's proliferative capacity at the meristem^12^ and root tip due to abundance of iron in acidic soil and red-light-induced activation of P uptake mediated by phytochrome-B^13^. Supplementary to this, a reduction in tissue P concentration critically affects plant growth, which leads to plant death^14^. Therefore, screening genotypes in P-starving conditions, understanding the significance of traits and their genetic mechanism, and the nature of heritability are important for developing plants tolerant of soil with low P.

Phosphorus efficiency was divided into two separate components: P-acquisition efficiency (PAE) and internal P-use efficiency (PUE)^15^. PAE refers to the uptake of P from the soil, and PUE is the use of taken-up P for plant growth and development. PUE and PAE in modern rice cultivars can be improved by using the low-P tolerance naturally found in landraces or wild species genotypes. Wissuwa et al.^16^ mapped a major QTL, *Pup1,* on chromosome 12 for tolerance of P deficiency from *aus*-type rice variety Kasalath. In addition to this, Chin et al.^17^ developed several markers for P-uptake efficiency to assist in breeding programs, among them, marker *OsPupK46-2* was particularly found associated with P-uptake efficiency. Gamuyao et al.^18^ termed *OsPupK46-2* as *phosphorus-starvation tolerance 1* (*PSTOL1*). *PSTOL1* promotes high phosphorus uptake by enhancing early root growth^18^. Globally, plant breeders are introgressing only the *PSTOL1* gene from Kasalath (*aus*) and African rice (*O. glaberrima* Steud.) to improve P uptake, which may narrow down the genetic variability of the *PSTOL1* gene. In addition, reports have mentioned QTLs associated with P deficiency in root and shoot traits at the seedling stage in *O. sativa* L.^19^. However, research in response to P deficiency and trait variability contributes to tolerance against low P and PAE and research on QTLs between wild and cultivated rice is limited. Wild species are a secondary gene pool that is a potential reservoir for unique genes uncommon in improved genotypes. Mapping and the identification of additional QTLs responsible for low-P tolerance with a large effect and pyramiding of those QTLs may give an additional level of tolerance under soil deficient in P along with yield improvement. This can be achieved by exploring new alleles for PAE and PUE.

Wild species of rice and landraces of the upland ecosystem may possess the required genetic resources. In addition to PAE and traits involved in low-P tolerance, P's microbial contribution by symbiotic interaction also plays an important role in providing P to plants in exchange for carbon^20^. By exploring the soil beyond what is reachable to roots and acquiring immobile P, fungal hyphae are efficiently involved in catering to the need of up to 80% of the P required by the plant^21–23^. The impact of mycorrhizae on P uptake under upland soil has been proved and associated candidate genes were reported^24^. However, the genetic basis of the response of rice cultivars in a panel of the population for arbuscular mycorrhizal (AM) fungal colonization under low-P conditions is not well established. Earlier reports on QTL identification for traits related to PAE and PUE are from bi-parental mapping populations. Reports on association mapping for low P tolerance and exploration at different species levels are not available in rice. Association mapping can simultaneously map several QTLs and serve as an excellent tool for allele mining by exploring natural variability in the germplasm. This study aimed to understand genetic variation in the level of tolerance among a panel of a population (*O. sativa,* improved varieties and landraces, and wild species, *O. nivara,* and *O. rufipogon*) containing 120 genotypes shortlisted from 155 through phenotyping to explore the distribution of *PSTOL1* across species and validated them under P-deficient conditions and identified QTLs for multiple traits (23) such as shoot and root system architecture, P accumulation, and colonization of AM fungi based on association mapping in response to low-P tolerance.

## Results

The present experiment began in order to explore the genetic variation and identification of QTLs associated with low-P tolerance in a unique population panel consisting of cultivated and wild relatives of *Oryza.*

### Phenotyping trait distribution pattern for adaptation to low P

**Figure 1:**
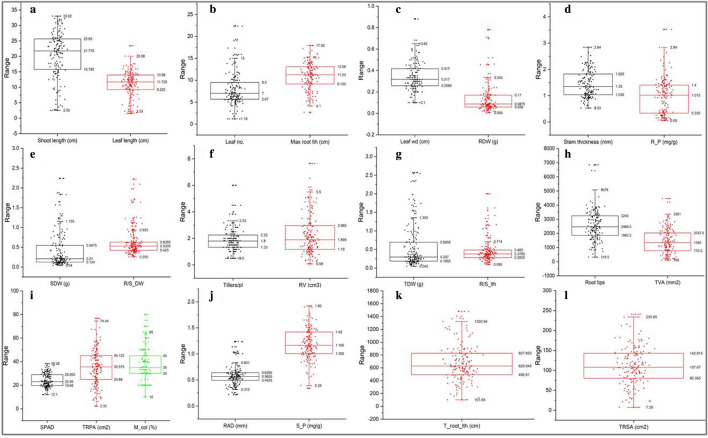
Box plot showing the population distribution of traits measured under P deficiency for 155 rice genotypes. **(a)** Shoot length and leaf length; **(b)** leaf number (leaf no.) and maximum root length (max root lth); **(c)** leaf width (leaf wd) and root dry weight (RDW); **(d)** stem thickness and root P content (R_P); **(e)** shoot dry weight (SDW) and root-shoot dry weight ratio (R/S_DW); **(f)** tiller number (Tiller/pl) and root volume (RV); (**g**) total dry weight (TDW) and root-shoot length ratio (R/S_lth); (**h**) root tips and top-view area (TVA); (**i**) SPAD, total root projected area (TRPA), and mycorrhiza colonization (%) (M_col); (**j**) average root diameter (ARD) and shoot P content (S_P); and (**k**) total root length (T_root_lth) and (**l**) total root surface area (TRSA). The upper, median, and lower quartiles of the boxes represent the 75th, 50th, and 25th percentiles of the population, respectively. The square box inside the quartile box represents mean and the asterisk represents outliers.

The frequency distribution of the population had a broad range of variation (Fig. 1). The mean and median were similar for most of the traits studied, and this indicates that the distribution frequency was normal. Among the various parameters studied, shoot length (−0.63), leaf length (–0.42), root length (−0.28), and shoot P content (−0.06) were negatively skewed, while the rest of the traits were positively skewed. The kurtosis values ranged from −0.63 to 8.71, the parameters were found to be > 3 TDW (total dry weight) (3.50), tiller number (3.69), shoot dry weight (3.87), root/shoot dry weight (6.89), root/shoot length (7.50), and root dry weight (8.71), indicating that the frequency distribution of the studied population for these traits was platykurtic. AM fungal root colonization ranged from 10% (improved genotypes) to 80% (landraces), while the average colonization percentage was significantly lower in improved genotypes (34%) than in landraces (41%) and wild genotypes (38%). The coefficient of variation (CV) of the entire population was reasonable and varied from 23% (SPAD) to 95% (root dry weight) (Table 1). The results obtained from the analysis of variance (ANOVA) revealed that significant (P < 0.0001) variation was observed among the genotypes for all 23 parameters studied (Table 1). The estimation of heritability (broad-sense) determines the insight into the extent of genetic control of the particular parameter and phenotypic accuracy to promote its breeding value. The estimated broad-sense heritability (h^2^) differed from 33.4% (maximum root length) to 74.8% (root P content) (Table 2). In addition, traits such as shoot dry weight, total dry weight, root-shoot length ratio, top-view plant area, total root length, root projected area, root surface area, root volume, mycorrhizal colonization, and root and shoot P content exhibited high h^2^ of > 60%.

**Table 1:** Analysis of variance, mean, range, and broad-sense heritability of 23 traits under P-deficiency conditions.

Traits	Mean	Minimum	Maximum	CV (%)	F ratio	P	H^2^ (%)
Shoot length (cm)	20.48	2.55	33.02	36	6.40	0.0001	54.7
Tillers plant^−1^	2.00	1.00	6.00	45	3.60	0.0001	42.4
Leaf number plant^−1^	8.00	1.00	22.00	46	8.79	0.0001	59.7
Leaf length (cm)	11.19	1.55	23.40	36	6.98	0.0001	56.2
Leaf width (cm)	0.34	0.10	0.88	38	5.70	0.0001	52.6
Stem thickness (mm)	1.44	0.53	2.84	35	3.72	0.0001	43.2
Max. root length (cm)	10.93	2.67	17.92	28	2.61	0.0001	33.4
SPAD	24.28	12.10	38.28	23	3.29	0.0001	40.1
Shoot dry weight (g)	0.39	0.04	2.24	89	20.21	0.0001	67.9
Root dry weight (g)	0.13	0.01	0.78	95	6.85	0.0001	55.9
Total dry weight (g)	0.53	0.05	2.57	91	16.72	0.0001	66.5
Root-shoot length ratio	0.45	0.10	2.00	67	13.08	0.0001	64.4
Total root length (cm)	675.85	101.65	1479.65	41	21.47	0.0001	68.3
Total root projected area (cm^2^)	35.54	2.32	76.69	41	11.42	0.0001	62.9
Total root surface area (cm^2^)	111.03	7.29	240.92	42	11.35	0.0001	62.9
Root avg. diam. (mm)	0.58	0.22	1.24	32	7.05	0.0001	56.4
Root volume (cm^3^)	2.23	0.08	7.65	65	10.28	0.0001	61.7
Root tips	2551.49	318.50	6846.00	40	7.38	0.0001	57.1
Root-shoot dry wt. ratio	0.63	0.26	2.22	55	6.13	0.0001	54.0
Top-view area (mm^2^)	1430.52	106.36	4470.78	61	25.11	0.0001	69.3
Mycorrhiza colonization (%)	37.26	10.00	80.00	38	259.88	0.0001	74.4
Shoot P (mg g^−1^)	1.20	0.34	1.92	24	201.29	0.0001	74.3
Root P (mg g^−1^)	0.95	0.05	3.52	69	872.82	0.0001	74.8

**Table 2:** Details of primers used for genotyping 120 rice genotypes and their estimated molecular genetic diversity parameters.

S. no	Marker	Allele no	Min. size of alleles (bp)	Max. size of alleles (bp)	Major allele frequency	Gene diversity (He)	Heterozygosity (Ho)	PIC	Shannon's index (I)	Nei's genetic diversity index (Nei)	Average heterozygosity
1	RM1112	3	100	110	0.601	0.487	0.020	0.378	0.714	0.488	0.460
2	RM1272	4	100	140	0.524	0.537	0.124	0.433	0.846	0.532	0.500
3	RM16	2	190	210	0.876	0.217	0.035	0.194	0.373	0.216	0.211
4	RM169	2	165	175	0.646	0.457	0.053	0.353	0.672	0.479	0.421
5	RM200	3	100	120	0.789	0.341	0.256	0.296	0.587	0.339	0.350
6	RM219	3	190	210	0.688	0.456	0.625	0.388	0.724	0.429	0.417
7	RM224	3	130	155	0.745	0.406	0.204	0.363	0.756	0.430	0.394
8	RM229	3	115	125	0.835	0.283	0.073	0.256	0.538	0.293	0.262
9	RM2334	3	130	200	0.471	0.565	0.291	0.468	0.932	0.577	0.543
10	RM235	3	125	140	0.573	0.551	0.227	0.471	0.914	0.563	0.458
11	RM237	3	125	135	0.797	0.332	0.052	0.292	0.570	0.316	0.299
12	RM242	3	195	230	0.753	0.393	0.021	0.346	0.696	0.408	0.370
13	RM254	2	150	165	0.991	0.018	0.000	0.018	0.045	0.015	0.011
14	RM259	2	150	160	0.767	0.357	0.008	0.294	0.547	0.361	0.343
15	RM26	2	100	120	0.885	0.204	0.010	0.183	0.350	0.198	0.177
16	RM261	2	130	135	0.902	0.177	0.000	0.161	0.496	0.316	0.244
17	RM28073	2	550	580	0.881	0.209	0.031	0.187	0.375	0.217	0.224
18	RM28102	3	160	180	0.775	0.366	0.112	0.326	0.637	0.352	0.349
19	RM283	3	145	160	0.845	0.275	0.176	0.257	0.574	0.297	0.265
20	RM287	3	100	125	0.722	0.418	0.085	0.354	0.693	0.416	0.372
21	RM297	4	140	180	0.525	0.570	0.238	0.484	0.984	0.577	0.527
22	RM30	4	70	100	0.569	0.572	0.413	0.503	1.025	0.572	0.537
23	RM306	2	155	170	0.806	0.313	0.012	0.264	0.526	0.343	0.310
24	RM3166	4	105	160	0.970	0.058	0.020	0.057	0.166	0.061	0.085
25	RM3295	4	80	120	0.505	0.585	0.263	0.502	1.003	0.590	0.560
26	RM3307	3	170	185	0.500	0.624	1.000	0.553	1.035	0.623	0.612
27	RM3343	2	140	150	0.626	0.468	0.748	0.359	0.652	0.459	0.383
28	RM335	5	100	150	0.505	0.642	0.476	0.585	1.215	0.655	0.631
29	RM3688	2	100	115	0.842	0.266	0.010	0.231	0.441	0.270	0.284
30	RM410	4	170	250	0.446	0.649	0.522	0.581	1.159	0.654	0.626
31	RM439	2	245	260	0.787	0.336	0.027	0.279	0.522	0.338	0.325
32	RM47	2	225	230	0.947	0.101	0.000	0.096	0.221	0.109	0.101
33	RM481	5	110	180	0.474	0.604	0.295	0.525	1.055	0.594	0.553
34	RM510	2	120	130	0.849	0.849	0.023	0.224	0.440	0.269	0.158
35	RM514	3	250	260	0.521	0.588	0.300	0.507	0.946	0.576	0.528
36	RM521	2	240	270	0.861	0.861	0.008	0.210	0.365	0.210	0.212
37	RM5349	3	110	125	0.583	0.506	0.052	0.402	0.776	0.509	0.477
38	RM5463	3	155	175	0.720	0.420	0.095	0.357	0.712	0.433	0.361
39	RM5485	2	140	160	0.945	0.103	0.000	0.098	0.199	0.095	0.059
40	RM553	2	155	165	0.754	0.371	0.026	0.302	0.548	0.362	0.333
41	RM574	2	150	155	0.828	0.285	0.031	0.244	0.451	0.278	0.258
42	RM5814	2	100	130	0.500	0.500	1.000	0.375	0.693	0.500	0.500
43	RM589	3	155	175	0.715	0.432	0.169	0.374	0.758	0.440	0.418
44	RM590	2	130	140	0.763	0.362	0.030	0.297	0.572	0.383	0.374
45	RM5926	3	110	160	0.818	0.312	0.106	0.286	0.603	0.319	0.288
46	RM6911	2	130	150	0.649	0.456	0.447	0.352	0.663	0.470	0.447
47	RM6966	3	140	180	0.663	0.468	0.071	0.387	0.776	0.478	0.476
48	RM7555	3	100	120	0.943	0.108	0.021	0.104	0.276	0.122	0.120
49	RM9	4	120	160	0.668	0.495	0.239	0.442	0.911	0.499	0.478
50	SSR12-17.4	2	450	480	0.788	0.334	0.047	0.278	0.519	0.336	0.330
51	K20-1	2	240	243	0.782	0.331	0.000	0.281	0.521	0.342	0.312
52	K20-2	2	982	995	0.606	0.478	0.000	0.364	0.693	0.500	0.500
53	K29-3	2	248	236	0.784	0.338	0.000	0.281	0.534	0.350	0.324
54	K29-1	2	206	212	0.630	0.466	0.043	0.357	0.641	0.449	0.328
55	PAP1	2	520	590	0.775	0.348	0.014	0.288	0.543	0.358	0.357
56	PAP3	2	260	500	0.539	0.497	0.713	0.373	0.690	0.496	0.493
57	PAP4	2	320	350	0.644	0.458	0.115	0.353	0.674	0.481	0.440
	Average	2.7	–	–	0.717	0.407	0.175	0.326	0.641	0.392	0.364

Additional primers evaluated: K41, K42, K43, K45, K46-1, and K46-2 (dominant markers); PAP5, RM142, RM164, RM184, RM24386, RM247, RM291, RM322, RM444, RM492, RM5314, RM536, RM538, SSR12-12.8, and ESSR12-20.2 (monomorphic primers).

**Figure 2:**
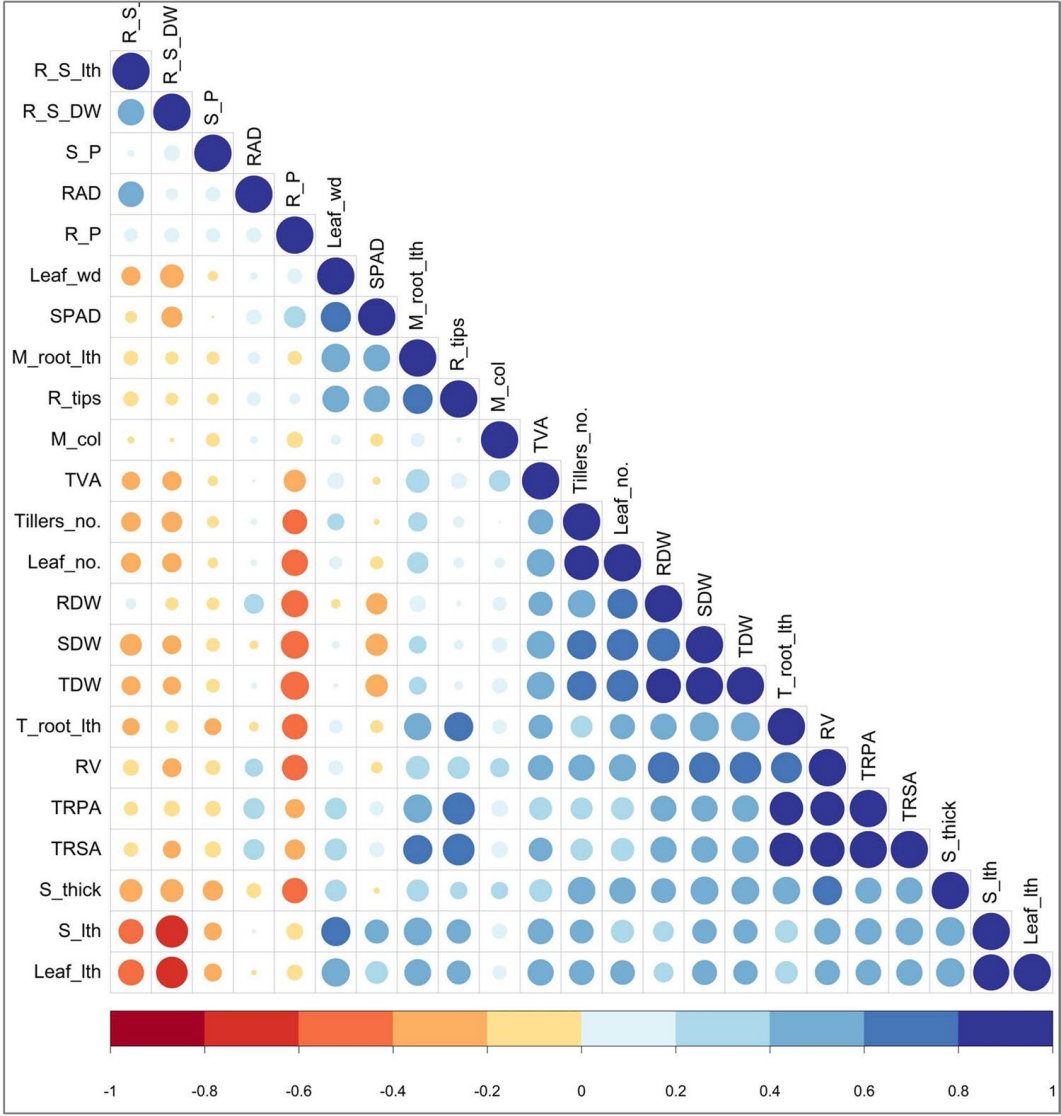
Pearson correlation matrix among the 23 traits measured under P deficiency for 155 rice genotypes. The color denotes the correlation, where 1 represents a complete positive correlation (dark blue) and −1 represents a complete negative correlation (dark red) between two traits. A large circle denotes strong correlation and a small circle indicates a weaker correlation.

Correlation analyses were executed to determine the correlation coefficient among the parameters associated with the traits responsible for low P in rice plants. Among the 23 traits studied, total dry weight had a positive correlation with 10 traits, followed by stem thickness, maximum root length, total root length, and total root surface area, which exhibited a positive association with eight traits (Fig. 2). In addition, a highly significant positive association was observed between shoot length and leaf length (r = 0.937), leaf number with tiller number (r = 0.8606), shoot dry weight (0.7066), and total dry weight (r = 0.7212). Similarly, shoot and root dry weight had a strong positive association between them. Also, the total root projected area exhibited a strong association with most of the root parameters, such as maximum root length (r = 0.8056), total root surface area (r = 0.9865), root volume (r = 0.8340), and number of root tips (r = 0.7378). The total root surface area also positively associated with root volume, root tips, and total root length. In contrast, root-shoot dry weight ratio exhibited a strong negative association with shoot length (r = −0.7486) and leaf length (r = −0.7114), wherein root P content also registered a negative association with shoot dry weight (r = −0.5524), root dry weight (r = −0.5198), and total dry weight (r = −0.5678).

**Figure 3:**
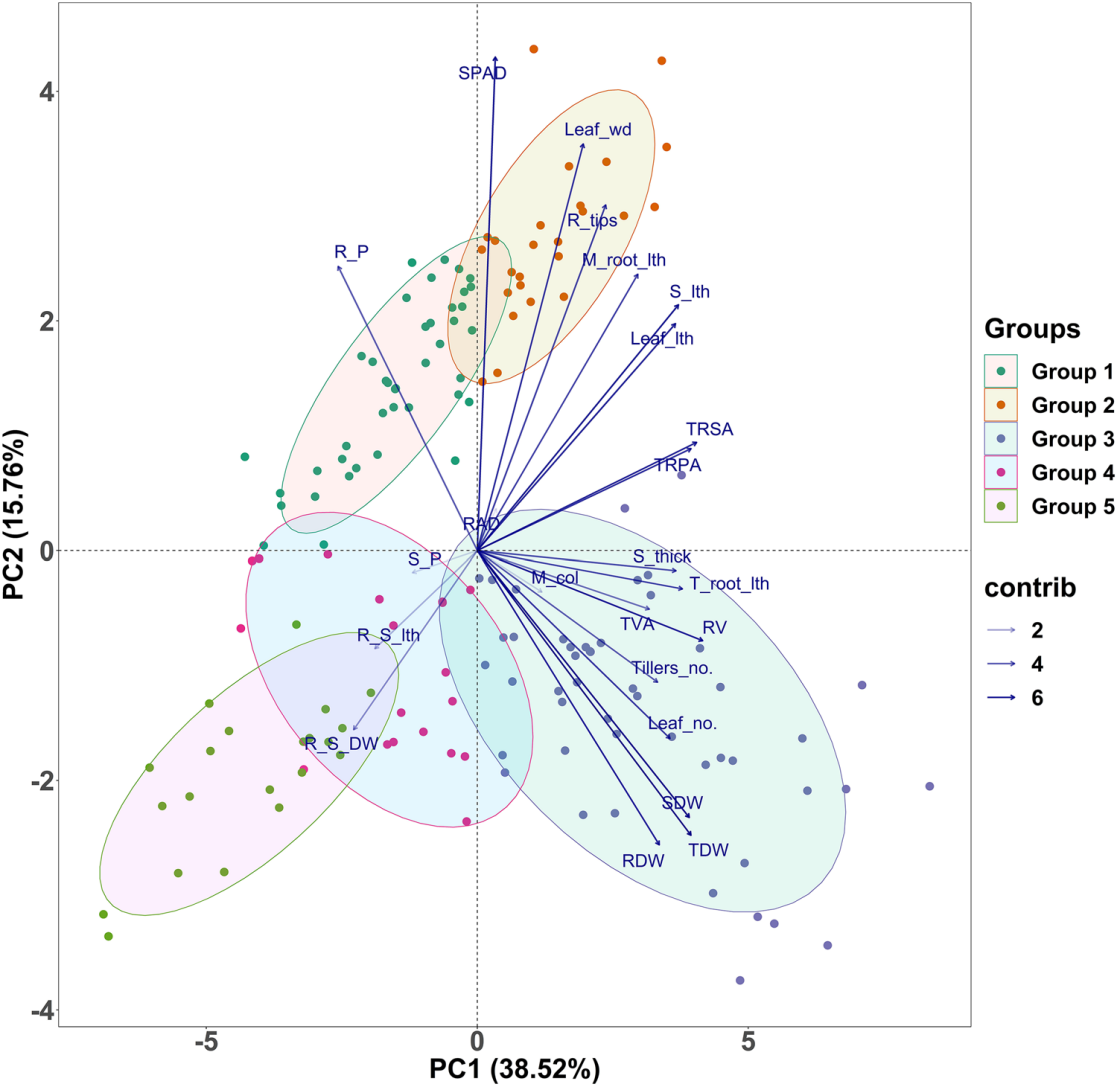
PCA biplot graph representing genotypes in two main principal components for traits measured under P-deprived conditions, and these two components explained 38.52% and 15.76% of the variance, respectively. The vector's direction and length indicate the traits' contribution to the first two components in the PCA. The transparency of the trait vectors represents the contribution to the variance in the dataset, ranging from 2% (lightest) to 6% (darkest). Genotypes were divided into five groups based on their level of tolerance. Groups 1 and 2 consisted of improved genotypes with high tissue P in root and more root growth, respectively. Group 3 consisted of the mixture of landraces, *O. rufipogon*, *O.nivara*, and positive checks. Group 4 consisted of a mixture of all species with more shoot P and Group 5 had *O. nivara* and *O. rufipogon* together with higher root-shoot dry weight ratio.

A multivariate principal component analysis was carried out to partition the traits associated with low P. A trait biplot analysis was composed and displayed for the first two principal components with a lay hold of 54.28% of the total variability depicted in Fig. 3. Principal component 1 (PC1) and PC2 accounted for 38.52% and 15.76%, respectively, toward the variability. Among the various traits studied, PC1 was strongly correlated with root- and shoot-related traits. PC1 increases with an increase in root volume, total root surface area, projected area, total root length, dry weight (shoot and root), stem thickness, and shoot and leaf length. It suggests that if root volume increases, the rest of the associated traits will increase, and genotypes would tend to have more root volume under P-starved conditions. PC2 had a positive association with SPAD and leaf width. However, root P content and root dry weight were negatively associated with PC1 and PC2, respectively. This suggests that the roots should translocate observed P and assimilate to expand the above-ground portion in genotypes under deficient P. Further, the biplot graph (Fig. 3) categorized the whole population into five major groups. Among these five groups, group 3 in quadrant 3 (bottom right) registered high biomass with low tissue P in shoot and root. On the contrary, group 2 in quadrant 2 exhibited more root growth with high root P. Other groups (1, 4, and 5) were presented on the opposite plane of the biplot and showed susceptibility under low P with high tissue P.

### Marker segregation and diversity index

A total of 78 primer pairs consisting of microsatellites and gene-based markers covering all 12 chromosomes were used to assess the genetic diversity of 120 genotypes. Fifty-seven of 78 (73.07%) primers used were polymorphic among the 120 selected genotypes. A total of 154 alleles were amplified with 57 polymorphic markers, with an average of 2.70 alleles per locus and the number of alleles ranging from 2 to 5 (Table 2). The amplification size of the markers ranged from 70 bp (RM30) to 995 bp (K20-2). The major allele frequency of the primers ranged from 0.446 (RM410) to 0.991 (RM254). In addition, genetic diversity (He) ranged from 0.018 (RM254) to 0.861 (RM521), with an average of 0.407. Heterozygosity (Ho) ranged from 0 (RM254, K20-2, K20-1, K29-3, RM261, RM5485, RM47) to 1 (RM3307, RM5814), with an average of 0.175. The PIC values of all 57 polymorphic primer pairs ranged from 0.018 (RM254) to 0.585 (RM335), with an average of 0.326. Shannon index (I) ranged from 0.045 to 1.215, with an average of 0.641, and Nei's genetic diversity index (Nei) ranged from 0.015 to 0.655, with an average of 0.392. The average heterozygosity estimated by POPGENE ranged from 0.011 to 0.631, with an average of 0.364.

### Analysis of molecular variance (AMOVA) and principal coordinate analysis (PCoA)

**Figure 4:**
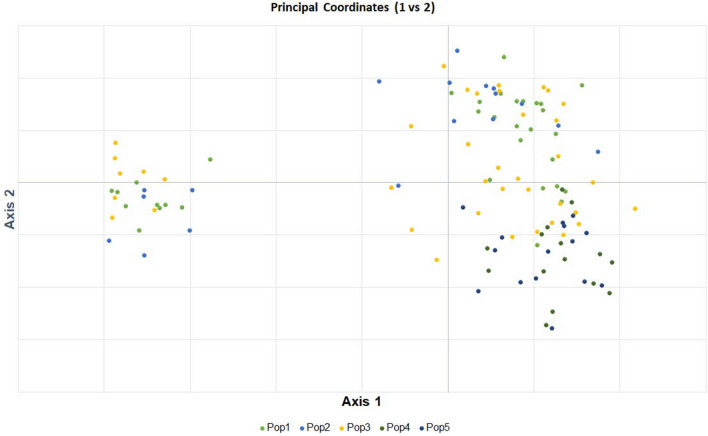
Principal coordinate analysis (PCoA) of the five sub-populations (Pop 1 (irrigated), Pop 2 (rainfed lowland), Pop 3 (upland), Pop 4 (*O. nivara*), and Pop 5 (*O. rufipogon*)) were plotted into three major clusters. **(a)** AMOVA showed maximum variation among the individuals, followed by within individuals and between populations. The genetic variability estimated by the fixation index revealed (Fst = 0.11) indicates the existence of moderate genetic differentiation within the population. **(b)** Nei genetic diversity among the assumed sub-population using principal coordinate analysis (PCoA). The assumed five sub-populations (Population 1 (irrigated), Population 2 (rainfed lowland), Population 3 (upland), Population 4 (*O. nivara*), and Population 5 (*O. rufipogon*)) were plotted into three major clusters.

In this study, 120 genotypes were divided into five populations based on their ecology and species level (irrigated, lowland, upland, *O. nivara,* and *O. rufipogon*) to determine the genetic differentiation among them. Maximum variation (71%) was found among the individuals, followed by within individuals (23%), and minimum variation existed among the populations (6%) (Table 3). The deviation from Hardy–Weinberg's prediction was calculated using Wright's F statistics. The F_IS_ and F_IT_ values for all the loci were 0.757 and 0.771, respectively, while F_ST_ was 0.056 between the populations. The NM value of the assumed population was 2.003. The PCoA explained that the first two components accounted for 20.97% of the total genetic variation among the assumed population (Fig. 4). Most of the genotypes were plotted on the right side of the plot, including wild species. However, all wild accessions from populations 4 (*O. nivara*) and 5 (*O. rufipogon*) were plotted in the separate quadrant on the plot's right side. Twenty-four accessions of populations 1 (irrigated; 10 in number), 2 (shallow lowland; 6 in number), and 3 (upland; 8 in number) were plotted on the extreme left side of the plot. Nei genetic distance congregated the five populations into three major clusters. Populations 4 and 5 were grouped into separate clusters, while populations 1 and 2 stayed together. The highest pairwise Nei genetic distance was noticed between population 1 (irrigated) and population 4 (*O. nivara*) (0.1158), followed by population 5 (*O. rufipogon*) with population 1 (0.1046) and population 2 (lowland) with 4 (0.1023). Minimum genetic distance was observed between populations 1 and 2 (0.0248), followed by that between populations 2 and 3 (upland) (0.0269).

**Table 3:** AMOVA between sub-populations and fixation indices of 120 genotypes for low-P tolerance in rice.

Source	df	Sum of squares	Mean sum of squares	Est. var	% of variation
Among populations	4	315.417	78.854	1.047	6
Among Individuals within populations	115	3543.646	30.814	13.278	71
Within individuals	120	511.000	4.258	4.258	23
Total	239	4370.063	–	18.583	100
F-statistics	Value	P (rand > = data)	–	–	–
Fst	0.056	0.001	–	–	–
Fis	0.757	0.001	–	–	–
Fit	0.771	0.001	–	–	–

### Genetic diversity and population structure

**Figure 5:**
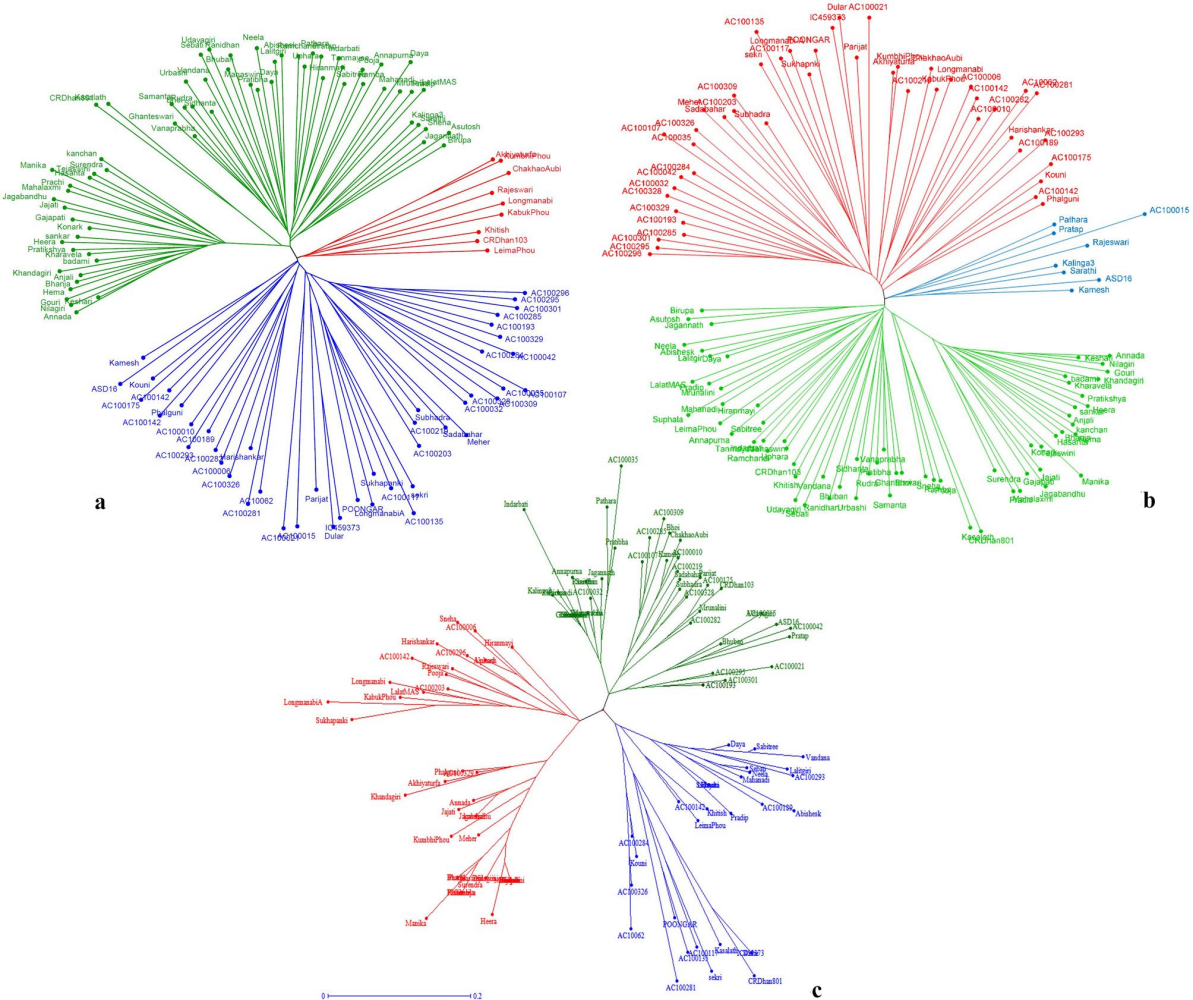
Unrooted tree using unweighted neighbor-joining (UNJ) method depicting clustering pattern of a panel population of 120 accessions in response to all 78 primers collectively, with and without *Pup1* markers. **(a)** 78 primers grouped genotypes into three major clusters. Cluster-I (blue) constitutes 45 genotypes that were divided into three major sub-clusters. Cluster-II (green) was divided into six sub-clusters with 65 genotypes, most of them being improved varieties, including CR Dhan 801 and Kasalath. Cluster-III (red) was grouped as a separate cluster with nine genotypes of northeastern states of India with two improved lines. **(b)** The cluster analysis with 65 low-P linked markers separated 120 genotypes into three groups. Cluster-I (green) represented only 65 improved lines Cluster-II (red) grouped 47 genotypes comprising wild species and a few *O. sativa* (landraces and improved lines) and Cluster-III (blue) had eight genotypes with seven improved varieties and one wild accession. **(c)** The cluster analysis with *Pup1-*specific markers grouped 120 genotypes into three major clusters. Cluster-I (red) consisted of 48 genotypes and was further divided into three sub-clusters. Cluster-II (green) separated 45 genotypes into three sub-clusters and Cluster-III (blue) represented 30 genotypes that were further divided into three sub-clusters with 14 (III-1), 13 (III-2), and 3 (III-3) genotypes. The positive checks Dular and Kasalath were grouped into sub-cluster III-2 with IC459373, multiple-stress-tolerant CR Dhan 801, Poongar, Sekri, Kouni, AC10062, AC100326, AC100284, AC 100281, AC 100135, and AC 100117.

Cluster and population structure analyses were carried out with the data generated with all 78 primers collectively, with and without 13 *Pup1*-specific markers. The cluster analysis based on unweighted neighbor-joining with 10,000 bootstraps with all the primers understudy grouped 120 genotypes into three major clusters (Fig. 5a). Cluster-I (blue) constitutes 45 genotypes divided into three major sub-clusters; all the wild cultivars fell into this cluster. Sub-cluster I-1 constitutes only two genotypes, Kamesh and ASD 16, followed by five genotypes (Kouni, AC100142, AC100175, Phalguni, and AC100010) grouped into Sub-cluster I-2. The rest of the 38 genotypes, including all the wild species and a few improved varieties (Meher, Sadabahar, Subhadra, Parijat) and landraces (Dular, IC459373, Sekri, Sukhapanki, Longmanabi A, Dular, and Harishankar), were grouped into Sub-cluster I-3. Cluster-II (green) was divided into six sub-clusters with 65 genotypes, most of which are improved varieties, including CR Dhan 801, whereas Cluster-III (red) was grouped as a separate cluster with nine genotypes (Akhiyaturfa, KumbhiPhou, ChakhaoAubi, KabukPhou, Rajeshwari, Longmanabi, Khitish, CR-Dhan103, LeimaPhou) of the northeastern states of India with two improved lines (Fig. 5a). Similarly, cluster analysis carried out with 65 low-P linked markers separated 120 genotypes into three groups (Fig. 5b). Cluster-I (green) represented only improved lines of 65 in number, whereas Cluster-II (red) grouped 47 genotypes comprising wild species and a few *O. sativa* (landraces and improved lines). Cluster-III (blue) had eight genotypes, with seven improved varieties and one wild accession.

Cluster analysis with 13 *Pup1*-specific markers grouped all 120 genotypes into three major clusters (Fig. 5c). Cluster-I (red) was further divided into three sub-clusters. A mono-genotypic cluster with genotype Hiranmayi was formed separately as Sub-cluster I-1, while Sub-cluster I-2 consisted of 15 genotypes with 12 *O. sativa* (landraces and improved varieties) and three wild accessions. In Sub-cluster I-3, 29 genotypes of improved varieties and landraces were grouped. On the other hand, Cluster-II (green) separated 45 genotypes into three sub-clusters: II-1 (10), II-2 (16), and II-3 (19). Sub-clusters II-2 and II-3 consisted of most of the wild accessions. Cluster-III (blue) represented 30 genotypes, which were further divided into three sub-clusters with 14 (III-1), 13 (III-2), and 3 (III-3) genotypes. Those with known low-P tolerance, such as Dular and Kasalath, were grouped into sub-cluster III-2 with IC459373, multiple-stress-tolerant CR Dhan 801, Poongar, Sekri, Kouni, AC10062, AC100326, AC100284, AC 100281, AC 100135, and AC 100117.

**Figure 6:**
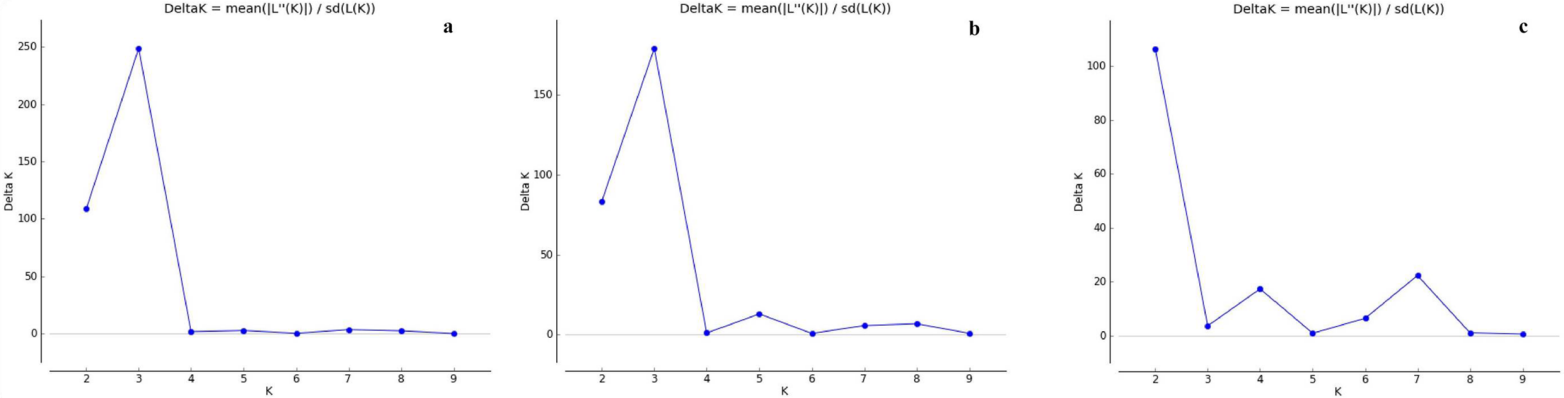
Graph of ∆K-value and ad hoc statistics related to the rate of change in the log probability of data between successive K-values. **(a)** 78 markers identified the highest log-likelihood with the number of populations set at three (K = 3) with ΔK = 248.51, **(b)** 65 markers linked to low P identified the highest log-likelihood with the number of populations set at three (*K* = 3) with ΔK of 179.01, and **(c)** *Pup1-*specific markers identified the highest log-likelihood with the number of populations set at two (*K* = 2) with ΔK = 106.29.

**Figure 7:**
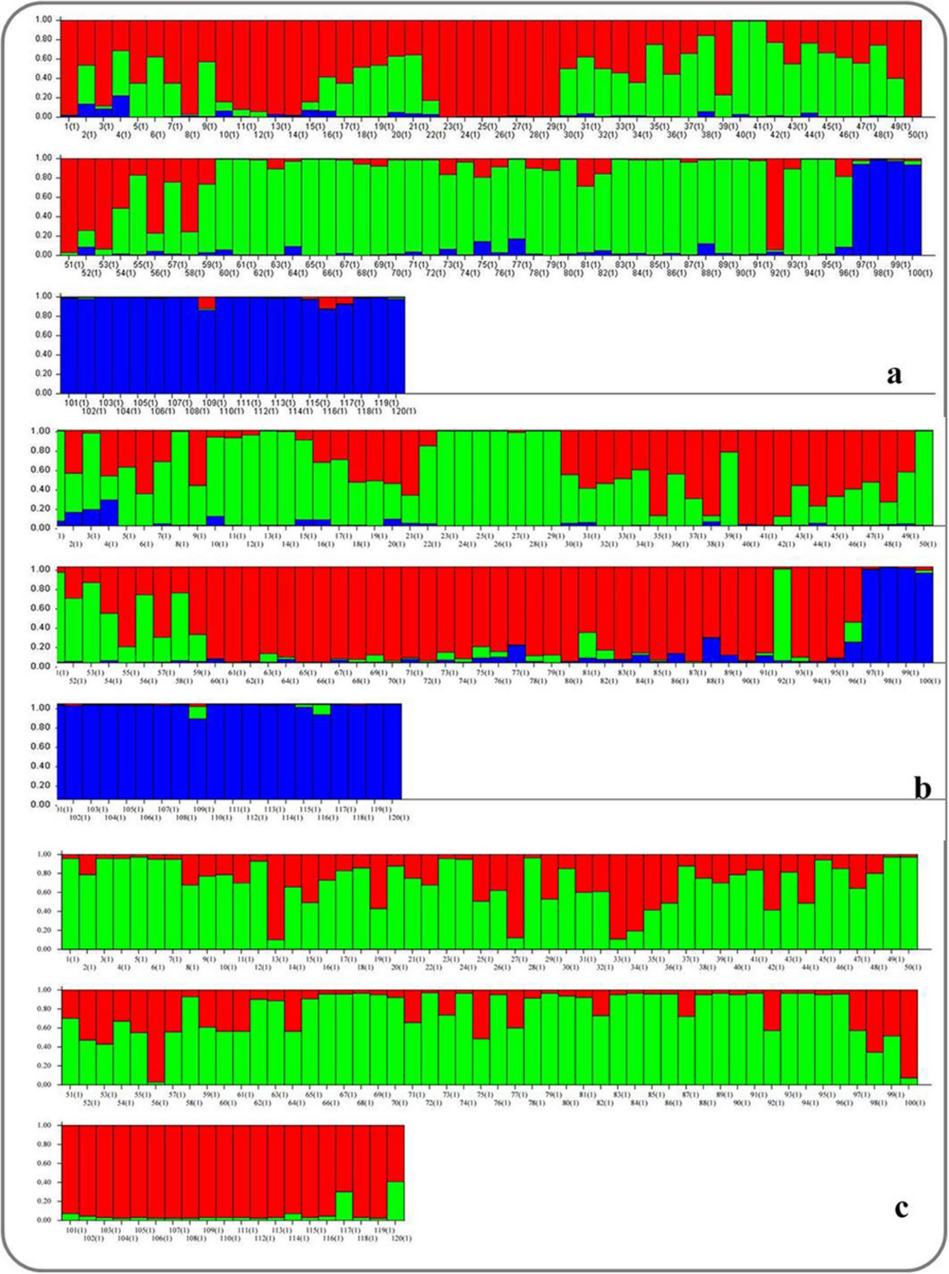
Distribution pattern of 120 rice accessions based on low-P linked markers and *Pup1*-specific markers determined by a model-based simulation, STRUCTURE 2.3.4. Grouping of accessions is based on **(a)** 78 markers, **(b)** 65 markers linked to low P, and **(c)** *Pup1-*specific markers. The number indicates the order of genotypes as mentioned in Table 1S.

Similarly, the genetic relationship among the genotypes was also determined by a model-based simulation, STRUCTURE 2.3.4, separately for all 78 markers under study, 13 low-P gene-specific markers (*Pup1*), and 65 primers without *Pup1* markers (Figs. 6a–c, 7a–c). The simulation, by using all 78 primers, identified the highest log-likelihood with the number of populations set at three (*K* = 3) (Pritchard et al. 2000). Structure harvester of Evano table (http://taylor0.biology.ucla.edu) analysis showed that, at K = 3, ΔK = 248.51, where the value was the highest in both independent burns (Fig. 6a). At K = 3, all 120 genotypes were divided into three sub-populations (SP). Among the 120 accessions studied, 25 accessions of wild species and landraces were placed in SP1, and 44 accessions fitted in SP2 comprising only improved genotypes. Similarly, 24 improved genotypes were grouped in SP3, and the rest of the wild species and landraces (27 genotypes) were grouped as an admixture (Fig. 7a). The fixation index (Fst) value ranged from 0.1880 in sub-population 1 of wild species to 0.5507 in sub-population 3 of improved fixed lines. The average distance (expected heterozygosity) was maximum (0.1261) between sub-population 1 of wild species and sub-population 3 of improved lines, whereas minimum distance was observed among sub-populations 1 to 2 (0.0797). The value of alpha (0.1270) reflects the relative admixture level between populations of less than 1, signifying origin mainly from one population (and each population is equally probable).

On the other hand, without a *Pup1*-specific marker, the 65 low-P linked markers identified the highest log-likelihood with the number of populations set at three (*K* = 3), with ΔK of 179.01 (Fig. 6b). Among the 120 genotypes, 46 genotypes of improved varieties were placed in SP1, and 24 genotypes fitted in SP2 comprising wild species and upland (landrace and improved) varieties. Similarly, 24 improved genotypes were grouped in SP3, and the rest of the wild species, landraces, and improved (26) accessions were grouped as an admixture (Fig. 7b). Fst values of the sub-populations are 0.2111 for sub-population 2 (wild and upland), 0.2760 for sub-population 1 (improved), and 0.5471 for sub-population 3 (improved). Allele frequency divergence between sub-populations 1 and 3 was maximum (0.1283), while minimum divergence was observed between sub-populations 1 and 3 (0.0768). The average distance (expected heterozygosity) between genotypes is high in population 2 (0.2863) and minimum with sub-population 3 (0.1623). The alpha value was nearer to zero (0.1226), suggesting that no admixture and population might have originated from one population.

The simulation using 13 *Pup1*-specific markers identified the highest log-likelihood with the number of populations set at two (*K* = 2), with ΔK = 106.29 (Fig. 6c). At K = 2, all 120 genotypes were divided into two sub-populations. Twenty-five genotypes of only improved varieties were placed in SP1, 63 genotypes fit in SP2 comprising improved varieties (including CR Dhan 801), wild species, and a few landraces (Dular, Kasalath, and IC459373), and the rest of the 32 genotypes of wild species and improved varieties were grouped as an admixture (Fig. 7c). The Fst value of sub-population 1 was 0.6382, and that of sub-population 2 was 0.3718. The allele frequency divergence among the two sub-populations was 0.2847, and the average distance (expected heterozygosity) among genotypes in the same sub-population was high in population 2 (0.2252) and minimum in population 1 (0.1548). The alpha value of the *Pup1*-specific markers is 0.3285, which signifies origin mainly in one population (and each population is equally probable).

### Association of markers with traits related to low P

In this study, both a generalized linear model (GLM) and mixed linear model (MLM) were used for association analysis at P-value < 0.005, FDR at the 5% level, and R^2^ > 7.5. In the GLM, 61 markers were associated with 110 QTLs (data not shown), and in MLM 16 markers were associated with 29 QTLs (Table 4). The R^2^ value of the associated markers for GLM ranged from 0.075 (RM283) to 0.190 (RM297), and for MLM it was from 0.076 (K29-3) to 0.149 (RM297). The marker F-value for GLM ranged from 7.95 (RM283) to 23.75 (RM297), and for MLM it ranged from 7.53 (K29-3) to 14.85 (RM297). A total of 19 QTLs for different traits were predicted on four chromosomes (1, 8, 11, and 12). A maximum of five QTLs (*qARD8.1, qLL8.1, qSL8.1, qTRP8.1,* and *qTSA8.1*) associated with average root diameter, leaf length, shoot length, total root projected area, and total surface area were identified on chromosome 8, followed by five QTLs (*qNT11.1, qRDW11.3, qRV11.3, qSDW11.2,* and *qTDW11.2*) found associated with tiller number, root dry weight, root volume, shoot dry weight, and total dry weight on chromosome 11 (Table 5). Five QTLs were harbored on chromosome 12, followed by four QTLs on chromosome 1, three on chromosome 3, and two (*qRL6.1* and *qTRL6.1*) on chromosome 6. In the MLM, nine of 23 traits under study were associated with more than one marker. The traits tiller number, root biomass (RDW), and root length were associated with a maximum of three markers on different chromosomes. Similarly, AM fungal root colonization (C%) was associated with two markers on chromosomes 2 and 4, with phenotypic variability (PV) ranging from 8.0 to 10.0%.

**Table 4:** Association of marker alleles with associated traits for low P in rice detected by MLM (Q + K) analysis in a set of 120 genotypes.

S. no	Trait	Marker	QTL name	Chromosome	Position (Mb)	*F-*value	*P-*value	*q-*value	R^2^
1	NL	RM283	*qNL1.1*	1	4.89	8.5916	0.0042	0.0238	0.0864
2	RDW	RM297	*qRDW1.1*	1	32.09	14.8585	0.0002	0.0214	0.1497
3	SDW	RM297	*qSDW1.1*	1	32.09	11.2506	0.0011	0.0192	0.1128
4	TDW	RM297	*qTDW1.1*	1	32.09	12.2008	0.0007	0.0245	0.1225
5	C%	RM521	*qMC2.1*	2	10.81	10.2264	0.0019	0.0211	0.1033
6	NT	RM2334	*qNT3.1*	3	26.55	9.8392	0.0023	0.0177	0.0994
7	RL	RM200	*qRL3.1*	3	13.40	8.6489	0.0041	0.0245	0.0872
8	TRL	RM200	*qTRL3.1*	3	13.40	8.8771	0.0036	0.0232	0.0896
9	C%	RM1272	*qMC4.1*	4	35.33	8.2245	0.0051	0.0224	0.0831
10	LW	RM574	*qLW5.1*	5	3.39	8.5373	0.0043	0.0232	0.0847
11	RL	RM30	*qRL6.1*	6	27.25	9.8689	0.0022	0.0189	0.0995
12	TRL	RM3343	*qTRL6.1*	6	29.10	8.4423	0.0045	0.0220	0.0852
13	ARD	RM6966	*qARD8.1*	8	27.32	8.4513	0.0045	0.0230	0.0844
14	LL	RM6966	*qLL8.1*	8	27.32	10.6437	0.0015	0.0194	0.1075
15	SL	RM6966	*qSL8.1*	8	27.32	7.6362	0.0068	0.0258	0.0771
16	TRPA	RM6966	*qTRP8.1*	8	27.32	7.9047	0.0060	0.0253	0.0799
17	TSA	RM6966	*qTSA8.1*	8	27.32	7.9043	0.0060	0.0243	0.0798
18	LL	RM242	*qLL9.1*	9	18.64	8.4178	0.0046	0.0213	0.0850
19	NT	RM242	*qNT9.1*	9	18.64	9.8677	0.0022	0.0206	0.0997
20	NT	PAP1	*qNT11.1*	11	2.43	7.5937	0.0070	0.0254	0.0767
21	RDW	RM5926	*qRDW11.3*	11	28.33	7.6993	0.0066	0.0260	0.0776
22	RV	RM5926	*qRV11.3*	11	28.33	12.3362	0.0007	0.0342	0.1246
23	SDW	RM5926	*qSDW11.2*	11	28.33	11.6389	0.0009	0.0240	0.1167
24	TDW	RM5926	*qTDW11.2*	11	28.33	11.0476	0.0013	0.0182	0.1109
25	RDW	K29-3	*qRDW12.2*	12	15.42	7.5396	0.0072	0.0236	0.0760
26	RL	K41	*qRL12.1*	12	0.26	7.5762	0.0071	0.0248	0.0764
27	SG	K20-2	*qSG12.2*	12	15.41	11.4432	0.0010	0.0210	0.1156
28	SG	K41	*qSG12.1*	12	0.26	8.9909	0.0034	0.0234	0.0908
29	TPA	K20-2	*qTPA12.2*	12	15.41	10.1975	0.0019	0.0194	0.1051

*SL* shoot length (cm), *NT* tillers plant^−1^, *NL* leaf number plant^−1^, *LL* leaf length (cm), *LW* leaf width (cm), *SG* stem thickness (mm), *RL* max. root length (cm), *SPAD,* *SDW* shoot dry weight (g), *RDW* root dry weight (g), *TDW* total dry weight (g), *TRL* total root length (cm), *TRPA* total root projected area (cm^2^), *TSA* total root surface area (cm^2^), *ARD* average root diameter (mm), *RV* root volume (cm^3^), *RT* root tips, *TPA* top-view area (mm^2^), *C%* mycorrhiza colonization (%), *SP* shoot P (mg g^−1^), and *RP* root P (mg g^−1^).

**Table 5:** Allelic variant of associated markers and favorable allelic and related phenotypic traits of the panel population.

Marker	Trait	Alleles	Favorable allele	Allelic variant			
				A	B	C	D
RM297	RDW	4	C(9)	0.08 ± 0.06ab	0.08 ± 0.06b	0.19 ± 0.23a	0.07 ± 0.09b
RM5926	RV	3	C(11)	0.36 ± 0.63b	1.70 ± 0.83a	1.91 ± 1.83a	–
RM297	TDW	4	C(9)	0.47 ± 0.43ab	0.33 ± 0.38b	0.87 ± 1.03a	0.34 ± 0.45b
RM5926	SDW	3	A(9)	0.63 ± 0.74a	0.25 ± 0.30b	0.23 ± 0.38b	–
K20-2	SG	2	B(90)	1.19 ± 0.61a	1.40 ± 0.70a	–	–
RM297	SDW	3	C(9)	0.39 ± 0.37ab	0.25 ± 0.32b	0.68 ± 0.79a	–
RM5926	TDW	2	A(9)	0.78 ± 0.91a	0.33 ± 0.36b	0.29 ± 0.45b	–
RM6966	LL	3	A(30)	11.03 ± 5.32a	10.21 ± 5.04a	1.00 ± 1.74b	–
K20-2	TPA	2	B(41)	1065.01 ± 832.38a	1311.65 ± 994.78a	–	–
RM521	MC	4	A(7)	50.28 ± 25.04a	37.88 ± 12.04ab	29.98 ± 18.01b	46.20 ± 22.39ab
RM242	NT	3	C(5)	1.43 ± 1.26b	1.55 ± 0.96b	2.86 ± 1.84a	–
RM30	RL	4	D63)	6.69 ± 5.22b	8.25 ± 5.51b	7.04 ± 4.07b	11.12 ± 11.18a
RM2334	NT	3	C(15)	1.44 ± 0.79b	1.36 ± 1.02b	2.01 ± 1.65a	–
K41	SG	1	A(50)	1.45 ± 1.20	–	–	–
RM200	TRL	3	C(4)	411.86 ± 309.84b	535.63 ± 287.45ab	816.25 ± 560.80a	–
RM200	RL	3	B(80)	7.67 ± 5.09b	10.44 ± 4.68a	9.19 ± 6.18ab	–
RM283	NL	3	B(97)	6.38 ± 6.40a	6.75 ± 3.45a	2.30 ± 3.22b	–
RM3343	TRL	2	B(82)	440.91 ± 484.40b	561.08 ± 234.64a	–	–
RM242	LL	3	C(5)	9.83 ± 7.81b	10.17 ± 5.39ab	12.61 ± 1.66a	–
RM574	LW	2	A(4)	0.44 ± 0.32b	0.35 ± 0.14b	–	–
RM6966	ARD	3	A(30)	0.47 ± 0.22b	0.45 ± 0.21a	0.06 ± 0.11b	–
RM1272	MC	4	A(3)	47.27 ± 41.62a	13.03 ± 20.52b	33.36 ± 14.87ab	29.59 ± 20.06ab
RM6966	TRPA	3	A(30)	30.26 ± 19.35a	28.09 ± 16.23a	–	–
RM6966	TSA	3	A(30)	95.06 ± 60.80a	88.27 ± 51.01a	5.33 ± 11.22b	–
RM5926	RDW	3	A(9)	0.14 ± 0.17a	0.07 ± 0.06b	0.06 ± 0.07ab	–
RM6966	SL	3	A(30)	19.97 ± 9.52a	18.83 ± 9.16a	2.47 ± 4.22b	–
PAP1	NT	1	A(53)	1.56 ± 0.61	–	–	–
K41	RL	1	A(51)	9.53 ± 5.85	–	–	–
K29-3	RDW	2	A(40)	0.10 ± 0.13a	0.07 ± 0.06b	–	–

Mean with ± is the standard deviation of the specific allele. Values with different alpabhet across allelic variant are significantly different with each other at p < 0.05. Values in parenthesis next to the favorable allele denotes the number of genotypes carrying that specific favorable allele.

*SL* shoot length (cm), *NT* tillers plant^−1^, *NL* leaf number plant^−1^, *LL* leaf length (cm), *LW* leaf width (cm), *SG* stem thickness (mm), *RL* max. root length (cm), *SPAD, SDW* shoot dry weight (g), *RDW* root dry weight (g), *TDW* total dry weight (g), *TRL* total root length (cm), *TRPA* total root projected area (cm^2^), *TSA* total root surface area (cm^2^), *ARD* average root diameter (mm), *RV* root volume (cm^3^), *RT* root tips, *TPA* top-view area (mm^2^), *C%* mycorrhiza colonization (%), *SP* shoot P (mg g^−1^), *RP* root P (mg g^−1^).

Among 29 QTLs observed in the MLM, 12 were associated with root parameters such as root dry weight (RDW), root length (RL), total root length (TRL), total surface area (TSA), average root diameter (ARD), total root projected area (TRPA), root volume (RV), and root biomass (RDW), with recorded PV from 10.32% to 14.97% on six chromosomes (1, 3, 6, 8, 11, and 12). Out of 16 markers associated with 29 QTLs, marker RM6966 on chromosome 8 had an association with five traits, LL, ARD, TRPA, TSA, and SL (shoot length), and explained PV of 7.7% to 10.7%. Similarly, RM5926 on chromosome 11, RM297 on chromosome 1, RM242 on chromosome 9, and K41 on chromosome 12 are associated with four, three, two, and two different traits, respectively, linked with low-P tolerance. Among the *Pup1*-specific markers, K20-2, K29-3, and K41 on chromosome 12 were associated with root dry weight, stem thickness, root length, and top-view area, with PV of 7.6% to 11.6%. The marker PAP1 developed from NRRI on chromosome 11 was associated with tiller number with a PV of 7.7%.

### Prediction of QTLs underlying candidate genes for traits associated with low-P tolerance

Twenty-nine QTLs related to traits measured under P starvation associated with 15 markers were considered to identify candidate genes (Table 6). Three markers, RM283 (chromosome 1), RM297 (chromosome 1), and RM242 (chromosome 9), associated with leaf number (*qNL1.1*), biomass (*qRDW 1.1, qTDW 1.1, qSDW 1.1*), leaf length (*qLL9.*), and tiller number (*qNT 9.1*) were found to have an association with growth promoter genes (auxin (*Os09t0491740*), brassinosteroid (*Os01t0178500*), and strigolactone (*Os01t0746400*)). Traits related to roots such as root length (*qRL6.1*), average root diameter (*qARD8.1*), root projected area (*qTRPA8.1*), and root surface area (*qTSA8.1*), associated with markers RM30 and RM6966, were found to be linked with candidate genes of phosphate starvation regulator (*Os06t0664800*) and phosphate transporter (*OsPT6, Os08g0564000*), respectively. On the other hand, tiller number (*qNT3.1* and *qNT11.1*) was associated with markers RM2334 and PAP1 on chromosomes 3 and 11, respectively, linked with locus *Os03t0672900* and *Os11t0149100* responsible for the regulation of plant growth and purple acid phosphatase (EC:3.1.3.2) involved in the acquisition and use of organic P.

**Table 6:** Co-localization of significant markers and candidate genes believed to be involved in low-P tolerance identified in the panel population.

Marker associated	Trait	QTL	LOC	Chr	Position	Description
RM283	NL	*qNL1.1*	Os01t0178500-02	1	4073916–4076438	Cross-talk of auxin and brassinosteroid signaling pathways, plant morphogenesis
RM297	RDW, TDW, SDW	*qRDW 1.1, qTDW 1.1, qSDW 1.1*	Os01t0746400-01	1	31225458–31228566	Control of lateral bud outgrowth, regulation of tillering, strigolactones biosynthesis, strigolactone and cytokinin controlled mesocotyl elongation in darkness
RM2334	NT	*qNT 3.1*	Os03t0672900-01	3	26576580–26579053	Cell wall deposition, regulation of plant growth and development
RM1272	C%	*qMC 4.1*	Os04t0688300-01	4	35207157–35208610	Haem peroxidase, plant/fungal/bacterial family protein
RM30	RL,	*qRL 6.1*	Os06t0664800-01	6	27456955–27459557	Phosphate starvation regulator
RM6966	LL, ARD, TRPA, TSA, SL,	*qLL 8.1, qARD 8.1, qTRPA 8.1, qTSA 8.1, qSL 8.1,*	Os08g0564000	8	28332207–28334033	Phosphate transporter (*OsPT6*)
RM242	LL, NT	*qLL 9.1, qNT 9.1*	Os09t0491740-01	9	18978153–18983471	Auxin efflux carrier domain-containing protein
PAP1	NT	*qNT 11.1*	Os11t0149100-01	11	2271010–2274026	Purple acid phosphatase (EC:3.1.3.2), improvement in phosphate acquisition and use

*SL* shoot length (cm), *NT* tillers plant^−1^, *NL* leaf number plant^−1^, *LL* leaf length (cm), *LW* leaf width (cm), *SG* stem thickness (mm), *RL* max. root length (cm), *SPAD, SDW* shoot dry weight (g), *RDW* root dry weight (g), *TDW* total dry weight (g), *TRL* total root length (cm), *TRPA* total root projected area (cm^2^), *TSA* total root surface area (cm^2^), *ARD* average root diameter (mm), *RV* root volume (cm^3^), *RT* root tips, *TPA* top-view area (mm^2^), *C%* mycorrhiza colonization (%), *SP* shoot P (mg g^−1^), *RP* root P (mg g^−1^).

The network analysis of these candidate genes in the QTL interval region was analyzed using the riceFREND database, and it revealed the coexpression pattern of the genes (Fig. 1S). A total of seven candidate genes were explored to construct gene networks, and their interactions were disposed of in Supplementary Fig. S1. Besides, the loci information and functions of the associated genes were given in Table 2S. These reported genes are functionally associated with physiological and molecular pathways in biotic and abiotic stress tolerance mechanisms and also uptake the nutrient element regulated by the phytohormone biosynthesis pathways. *Insilico* expression analysis of these genes indicates that most genes are highly expressed in root tissues based on the RiceXPro database. The sum of nodes and edges of coexpressed genes of the seven-candidate genes were 72 and 134 respectively. Among the seven putative candidate genes, the locus *Os08g0564000* located on chromosome 8 had functionally associated with eight known genes (*OsRLCK266, OsWRKY32, OsRAM2, OsbHLH023, OsCP25, OsNPC4, OsERD6*, and *OsCP29*). Similarly, other locus had functional associated with genes related to root growth and auxin.

### Identification of rice germplasm with QTLs related to low-P tolerance traits such as root growth and shoot and root biomass

A total of seven rice accessions were found to tolerate P-deficient conditions, including tolerant checks Dular and Kasalath. Among them, the genotypes IC459373, Chakhao Aumbi, AC100219, AC100062, and Sekri exhibited a par or higher shoot and root dry weight with better root growth than the checks in P-starved conditions. Genotype IC459373 outperformed the checks, while Chakhao Aumbi and AC100219 performed significantly better under low P. Amid the favorable QTLs detected, six QTLs (Table 7) were found in Sekri, followed by five QTLs detected in Chakhao Aumbi having a high tiller number and biomass. The genotypes IC459373 and Kasalath had four QTLs each. Therefore, these accessions could be used as donors in a breeding program to improve PUE in rice.

**Table 7:** List of genotypes identified with their QTL details and reaction against P deficiency in rice.

S. no	Genotype	SL	NT	RL	SDW	RDW	TRPA	TSA	RV	QTLs
1	Dular	25.93	3.50	11.55	1.220	0.270	54.72	171.91	4.25	*qTRL 6.1, qLL 9.1, qNT 11.1*
2	Kasalath	26.75	3.00	12.33	1.246	0.268	33.48	105.18	1.48	*qRDW 1.1, qTDW 1.1, qSDW 1.1, qNT 11.1*
3	IC459373	28.93	3.33	15.12	1.459	0.278	57.36	180.20	2.78	*qNT 3.1, qTRL 6.1, qLL 9.1, qNT 11.1*
3	ChakhaoAumbi	29.22	3.00	10.20	1.870	0.260	44.24	138.98	4.20	*qNL 1.1, qRDW 1.1, qTDW 1.1, qSDW 1.1, qNT 3.1*
4	AC100219	26.10	3.33	16.30	1.570	0.290	54.44	133.85	6.53	*qNT 3.1, qTRL 6.1, qNT 11.1*
5	AC100062	26.23	3.00	10.32	1.350	0.310	35.84	112.61	4.33	*qNL 1.1, qNT 3.1, qTRL 6.1*
6	Sekri	30.48	3.17	11.62	1.120	0.300	50.29	157.99	5.50	*qRDW 1.1, qTDW 1.1, qSDW 1.1, qRL 6.1, qLL 9.1, qNT 11.1*
7	Abhishek	14.48	1.83	7.42	0.100	0.040	6.30	19.78	0.30	*qNT 11.1*
8	AC100282	9.05	1.00	4.40	0.04	0.01	2.32	7.29	0.08	–
9	Jagabandhu	15.10	1.50	5.15	0.065	0.020	8.14	25.57	0.33	–
10	Kanchan	17.85	1.50	6.27	0.082	0.021	12.97	40.75	0.53	–
11	Hiranmayi	17.78	1.00	9.47	0.051	0.023	13.63	42.83	0.83	–
12	Parijat	15.12	2.00	9.17	0.084	0.027	16.41	51.55	0.74	–

*SL* shoot length (cm), *NT* tillers plant^−1^, *NL* leaf number plant^−1^, *LL* leaf length (cm), *LW* leaf width (cm), *SG* stem thickness (mm), *RL* max. root length (cm), *SPAD, SDW* shoot dry weight (g), *RDW* root dry weight (g), *TDW* total dry weight (g), *TRL* total root length (cm), *TRPA* total root projected area (cm^2^), *TSA* total root surface area (cm^2^), *ARD* average root diameter (mm), *RV* root volume (cm^3^), *RT* root tips, *TPA* top-view area (mm^2^), *C%* mycorrhiza colonization (%), *SP* shoot P (mg g^−1^), *RP* root P (mg g^−1^).

## Discussion

Researchers globally for long been trying to understand the critical role of P in plant growth and development mediated through signaling and metabolism to develop P-efficient cultivars in several crops. Considering the area occupied and the importance of rice, improving PUE is catching the attention of rice researchers. Because of finite P fertilizer and its availability only in specific regions around the globe^4^, the applied P fertilizer becomes unavailable to plants because of high reactivity with soil particles and microbial activity^25,26^. Exploring the large unavailable form of soil-bound P would be an alternate strategy to mitigate these factors. In addition, increasing the tolerance level through PUE, acquiring P from the soil by exploring the adjacent area and by modifying the root architecture, secretion of root exudates, and symbiotic association with AM fungi would be advantageous. Attempts were made to improve P efficiency in rice through classical plant breeding by transferring targeted traits with limited success^27^. The available germplasm in gene banks has sufficient genotypic variation to improve P efficiency, but the major obstruction in identifying and developing P-efficient genotypes is the lack of a screening facility with low-P soil. Predominantly, the identification of P-efficient genotypes is driven by biomass production of shoot or root under P-deficient soil. To overcome this impediment, QTLs/genes related to traits that improve P-use efficiency need to be identified to introgress them into an elite background. Wild species and landraces possess higher genetic diversity than modern/improved varieties, which serve as a reservoir of genetic diversity and help to use the beneficial alleles in breeding programs for crop development. This research work attempted to study the genetic diversity, structure, and association between markers and traits measured under low-P conditions among the selected subset of the population comprising wild species, landraces, and improved varieties with multiple traits.

### Evaluation and identification of genotypes with tolerance to low P

**Figure 8:**
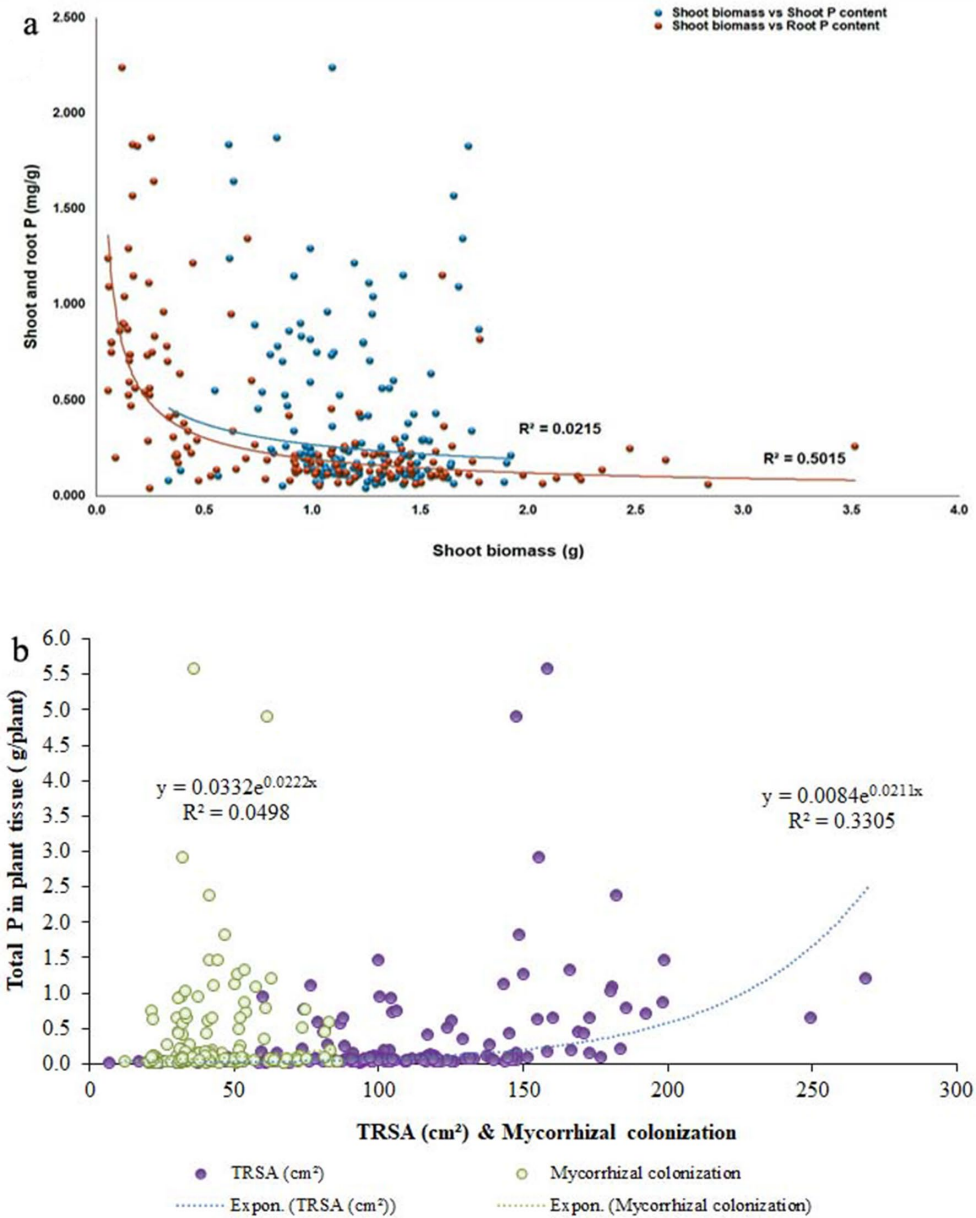
Relationships between shoot biomass, total root surface area (TRSA) and mycorrhizal colonization with P under deficient condition. **(a)** The negative association between shoot biomass and root P concentration suggests that an increase in biomass (shoot) in deprived P was associated with the dilution effect of P. **(b)** The line indicates the fitted results representing the relationship between total P of plant tissue and possible parameters (TRSA and mycorrhizal colonization) involved in P uptake under P deficient condition. The contribution of improved P uptake of TRSA was high compared to mycorrhizal colonization.

**Figure 9:**
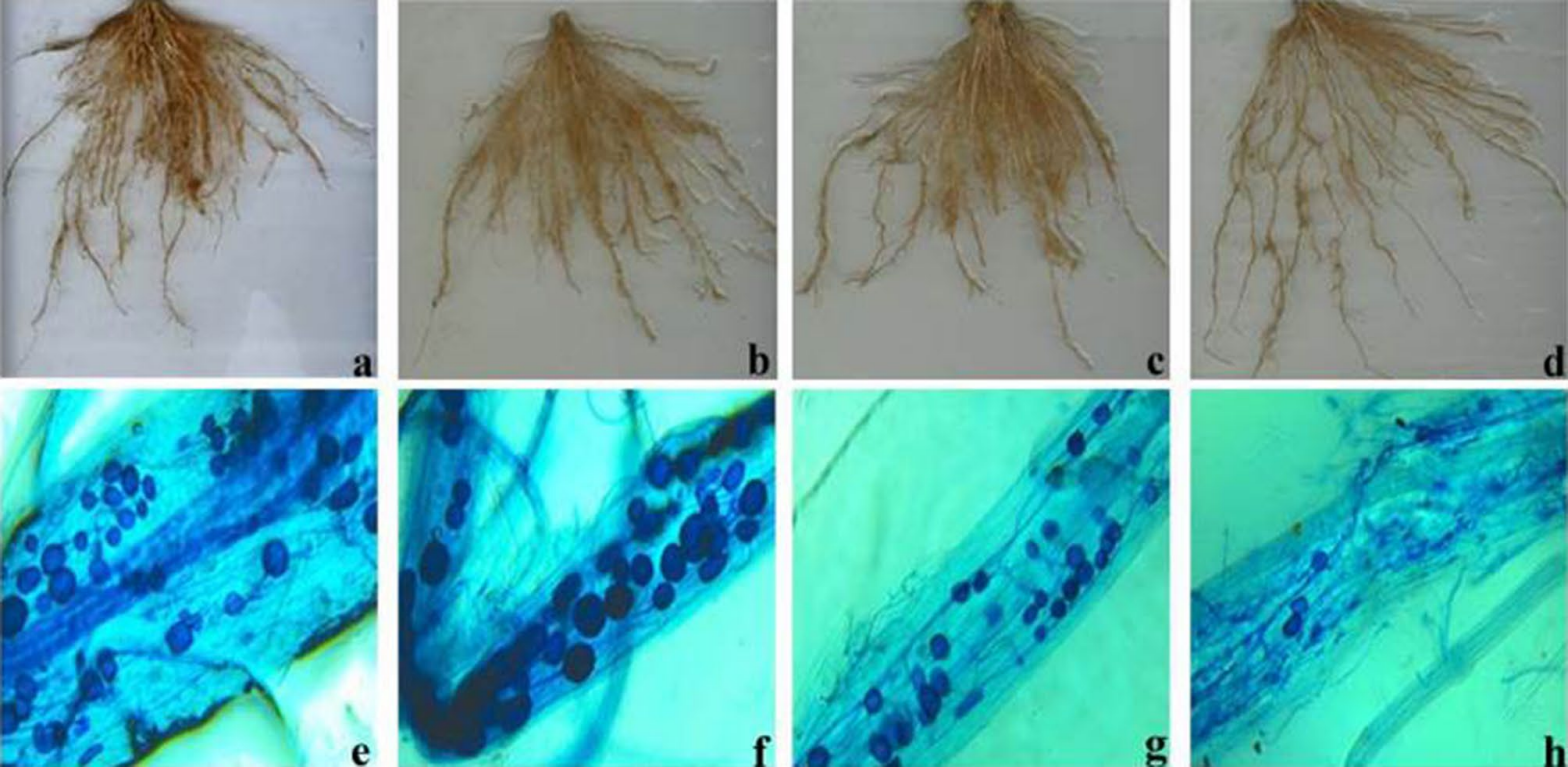
Root and AM colonization in different rice genotypes found in low-P soil as shown by trypan blue staining. **(a,e)** Dular, **(b,f)** Kasalath, **(c,g)** Sekri, and **(d,h)** AC100219.

This study included accessions of improved genotypes, landraces, *O. nivara*, and *O. rufipogon* from nine provinces of India. The frequency distribution and CV exhibited substantial variability, and adaptation to P starvation was observed among the 155 accessions of *Oryza* species. Similarly, ANOVA revealed the presence of significant variation among genotypes for all the parameters observed. In addition, traits such as biomass and tissue P measured under low P registered a high h^2^ of > 67%. Similarly, high heritability of 81% to 91% was reported by Wang et al.^28^ for total above-ground biomass, total above-ground P uptake, and P translocation efficiency in P-starved conditions. Therefore, the presence of moderate to high genetic advance with high h^2^ offers an opportunity to improve the selection of traits at the early generation based on shoot biomass, total root length, tissue P content, mycorrhizal colonization, and the geometric trait of top-view area for improving low-P tolerance in rice. The strong positive correlation between shoot and root biomass and inter-correlation among the root parameters show the possibility of improving P-efficient genotypes. The negative association between root P content and shoot dry weight (Fig. 8a) suggests that genotypes with desired root architecture QTLs such as *Pup1* coupled with high-affinity P transporters (*PHT1* family) would be supportive of using available P efficiently in P-deprived soil^14^. Besides, TRSA had become the higher contributor for improved P uptake under deficit P than mycorrhizal colonization. This was clear from Fig. 8b, and non-linear regression showed that 33% of P uptake would be through TRSA, while the role of mycorrhizae in P uptake was minimum (4%) in the present experiment. The multivariate principal component analysis also suggests the importance of roots under P-starved conditions to improve shoot growth. Genotypes such as upland landraces of northeast India, a few genotypes of improved upland varieties, *O. nivara*, *O. rufipogon,* and known tolerant genotypes Dular and Kasalath were categorized under group 3 with high biomass and low tissue P in both shoot and root, whereas genotypes of the improved varieties were put in groups 1 and 2 with high tissue P in the root and low P in the shoot. Conversely, *O. nivara* and *O. rufipogon* were grouped together (group 5) with high tissue P, root diameter, root-shoot weight ratio, and root-shoot length ratio, while group 4 had a mixture of all species with more shoot P. This suggests that genotypes of group 5 (*O. nivara* and *O. rufipogon*) can extract, upload, and translocate P from the rhizosphere to the shoot in low-P soil. The symbiotic association between AM fungi and plants (Fig. 9) was significantly higher in groups 3 and 2 (41%), in which they have a good root system (Fig. 3). The improved genotypes of group 1 had minimum colonization of 30% with a poor root system. It is well-known that rice plants with more root biomass positively correlate with AM fungal root colonization^29^.

### Genetic diversity and distribution of *Pup1* among *Oryza* spp.

Elucidating molecular genetic diversity helps breeders estimate rice germplasm's genetic constitution and select donors to develop a systematic and effective breeding program. Fifty-six of 78 markers exhibited polymorphism (73%), which gave rise to 154 alleles, less than in our previous study^30^. We obtained 128 alleles from 39 polymorphic primers, and more than reported by Donde et al.^31^, 154 alleles from 65 polymorphic markers. The present average of 2.7 alleles per locus was lower than that of many other research reports; for example, Noyer et al.^32^ analyzed 419 rice accessions using 16 SSR markers and reported an average of 9.1 alleles per locus. The higher average number of alleles per locus than in the present study (2.24) may be due to the use of a larger number of genotypes. This study's average PIC value was 0.326, which was a bit higher than that of Xu et al.^33^ (0.31), who studied the association mapping of cold tolerance in improved *japonica* rice germplasm.

Cluster analysis based on unweighted pair group method with arithmetic mean (UPGMA) with all 78 markers and excluding gene-specific *Pup1* grouped 120 genotypes into three clusters. Under both conditions (excluding and including the *Pup1*), the wild species were grouped into a single cluster and might have a similar type of P-efficiency mechanism under P deficiency. However, all explored wild species (30 in number) might have lower diversity because they share common geographical boundaries such as Odisha and neighboring province West Bengal of India. This coincides with several studies on assessing genetic diversity^34–36^. On the other hand, those wild species were separated from *O. sativa* L. (landraces and improved genotypes). Similarly, Xu et al.^33^ studied the genetic diversity in 103 rice germplasm accessions comprising *O. rufipogon* and *O. sativa* genotypes, and grouped both of them in separate clusters, which are in support of our study. The UPGMA cluster analysis with 13 gene-specific *Pup1* markers grouped all 120 genotypes into three major clusters (Fig. 5c). Cluster-III (blue) grouped 25% (30 accessions) of the studied population, including the tolerant genotypes Dular and Kasalath having *Pup1*. However, this cluster again sub-divided into three sub-clusters (III-1 (14 in number), III-2 (13 in number), and III-3 (3 in number)) based on the presence of gene-specific *Pup1* markers. Among the three sub-clusters, sub-cluster III-2 had grouped 13 genotypes that include six landraces (42.85% of the total population), five *O. rufipogan* (33.3%), one *O. nivara* (AC 100117) (6.66%), and one improved variety (CR Dhan 801) (1.31%). The performance of those 13 genotypes registered 28.42% shoot length, 83.41% root biomass, and 90.49% root volume, with 35.88% top-view area, higher than in the genotypes secured in the other two sub-clusters. On the other hand, out of 13 gene-specific *Pup1* markers, K29-3, K41, K43, and K45 played an important role in differentiating genotypes between these sub-clusters. To corroborate this, Chin et al.^17^ reported K29-1, K29-3, K41, K43, K45, and K46-1 as core markers to differentiate genotypes that differ in *Pup1* allele constitutions. Further, Neelam et al.^37^ substantiated that *O. rufipogon* collected from India has high PUE with a differential functional allele of *PSTOL1*.

By considering all 78 markers and excluding gene-specific *Pup1* markers, the model-based simulation STRUCTURE 2.3.4 divided the population into three sub-populations, similar to the distance-based model. Conversely, the gene-specific *Pup1* markers grouped the population into two sub-populations. The grouping of genotypes with distance and model-based analysis was similar at the genetic level with markers linked to low P, including exclusive markers or exclusive gene-specific *Pup1* markers. It suggests that traits other than early root growth (*Pup1-*specific markers) in differentiating low-P tolerance must be documented. AMOVA revealed a higher proportion of variation among the individuals, while a lower proportion of variation was observed among the populations. A similar observation of a higher proportion of variation among the individuals was reported by Verma et al.^38^ and Islam et al.^39^. Genotypes of the present study belonged to different species of *Oryza* (*sativa, rufipogon,* and *nivara*), wherein the species *sativa* includes genotypes of landraces and from improved upland, lowland, and irrigated ecosystems, which resulted in a higher proportion of variation among the individuals than among the populations. Within-individuals that had registered higher variation indicated a high level of heterozygosity at each locus in wild genotypes and landraces. The Fst value was minimum in the case of sub-populations containing wild genotypes (0.20), while a high Fst index of 0.55 was observed in improved genotypes, suggesting that they are fixed and homozygous in nature. The alpha value determined by the model-based simulation STRUCTURE 2.3.4 under three circumstances was nearer to zero (0.12) in two instances, which suggests that the individuals are from one population, while the *Pup1* gene marker represented with 0.3285 suggests that the variation arising in a specific region might be variation in the *Pup 1* indel region. The value of NM (2.0) indicated possible gene flow between the populations and low genetic differentiation for the trait studied^40,41^. Therefore, a hypothesis has been proposed that the *Pup1* region or gene with low-P tolerance might have been introgressed from *O. rufipogon* into *O. sativa*. This may be supported in this study by the frequency of tolerant accessions of *O. rufipogon* outnumbering those of *O. nivara*. Further, Neelam et al.^37^ substantiated that *O. rufipogon* (IRGC 106506) accessions performed significantly better under limited P, with 2.5 times higher root weight than the positive control.

**Figure 10:**
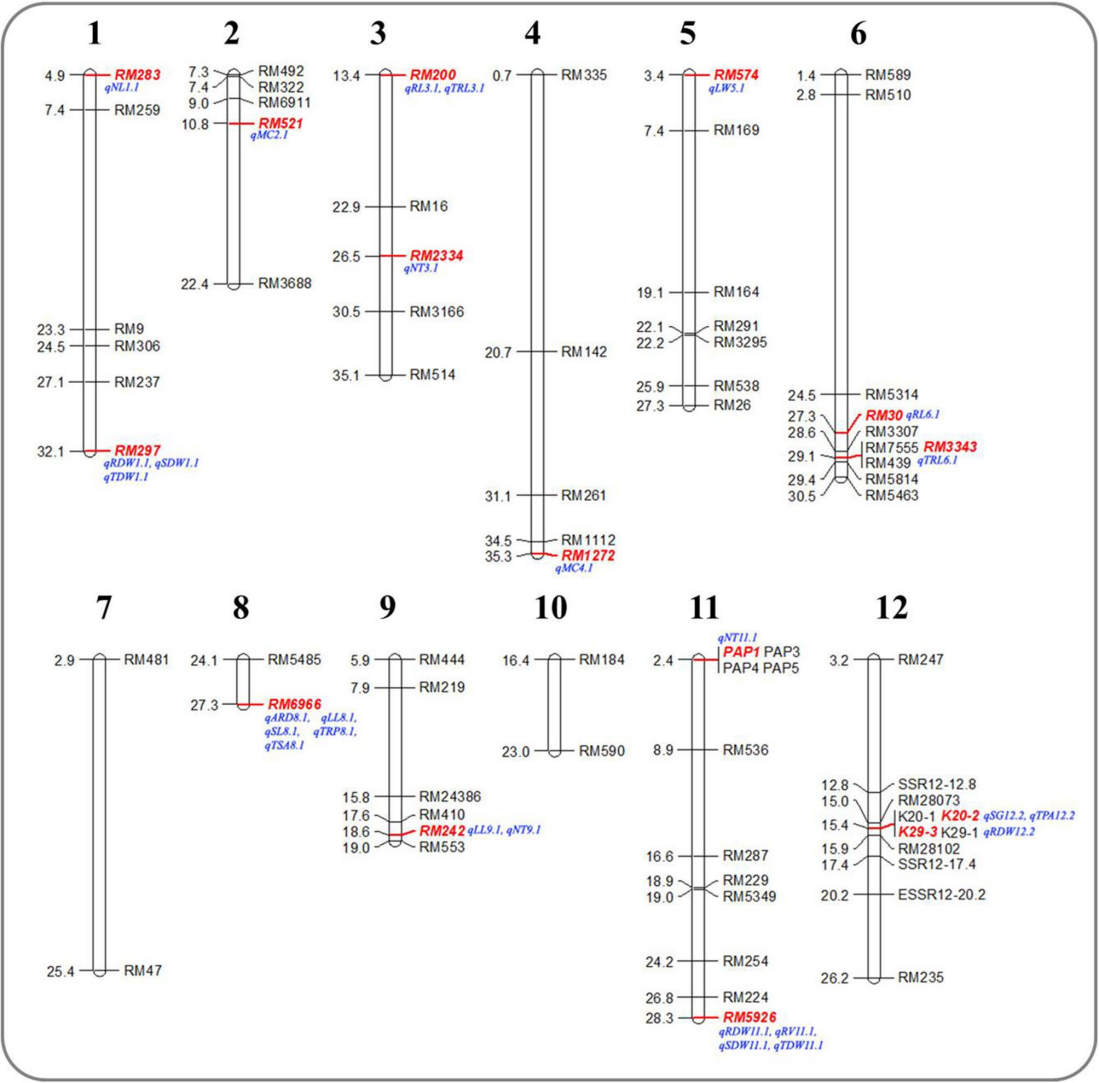
Distribution of primers used for association mapping and detected QTLs on 11 chromosomes of rice. Distances on the map are in Mbp presented on the left-hand side of the chromosomes. Markers highlighted in red are found associated with adaptive traits under P-deprived conditions. [*SL* shoot length (cm), *NT* tillers plant^−1^, *NL* leaf number plant^−1^, *LL* leaf length (cm), *LW* leaf width (cm), *SG* stem thickness (mm), *RL* max. root length (cm), *SPAD, SDW* shoot dry weight (g), *RDW* root dry weight (g), *TDW* total dry weight (g), *TRL* total root length (cm), *TRPA* total root projected area (cm^2^), *TSA* total root surface area (cm^2^), *ARD* average root diameter (mm), *RV* root volume (cm^3^), *RT* root tips, *TPA* top-view area (mm^2^), *MC* mycorrhiza colonization (%), *SP* shoot P (mg g^−1^), *RP* root P (mg g^−1^)].

In the present association study, both GLM and MLM were used to assess the association between traits and markers. However, the number and degree of association-related traits are based on the dataset and model used. MLM, unlike GLM, is more accurate and has a robust algorithm to improve the calibration. It integrates structure and kinship matrix, which rectifies the false-positive error expected due to population structure and relatedness^42^. Hence, MLM has been popularly used by several researchers for studying marker-trait association. In the GLM, 61 markers were associated with 110 QTLs; in the MLM, 16 markers were associated with 29 QTLs. The total number of QTLs and distribution of the markers are displayed in Fig. 10. The unique population exploited in this study was associated with 78 markers linked to low P with multiple traits screened under deficient P. Not much study was carried out on the traits selected in this study in reference to the association with tolerance of P deficiency. This resulted in the identification of novel and interesting QTLs for multiple traits. We reported 16 markers (RM6966, RM1272, RM200, RM2334, RM242, RM283, RM297, RM30, RM3343, RM521, RM574, RM5926, PAP1, K20-2, K29-3, and K41) associated with 29 QTLs for 17 traits (ARD, C%, LL, LW, NL, NT, RDW, RL, RV, SDW, SG, SL, TDW, TPA, TRL, TRPA, and TSA) under low-P conditions in rice. Overlapping of markers for tolerance of low P suggests a high correlation between the traits. For instance, marker RM6966 was found to be associated with several root-related traits necessary for low-P conditions. Similarly, RM242 and RM297 were linked to shoot and root biomass. Thus, the pleiotropic effect of markers unravels the genetic correlation among the traits^30^ measured under low P. Marker RM30 on chromosome 6 was observed to be significantly associated with root length (R^2^ = 9.9%), and was co-localized with a QTL reported for root elongation ratio under P deficiency with PV of 19.9%. The tolerance imparted by this QTL might be due to the positive regulation of the candidate gene *Phosphate starvation regulator* (*Os06t0664800*) at 0.15 Mb to the right of *qRL6.1* on chromosome 6. The network analysis of locus *Os06t0664800* by the riceFREND database revealed that it has 11 nodes and 19 edges. It was found that coexpression patterns of the genes were highly expressed in roots, specifically in protophloem sieve elements and promote root elongation (*OsbHLH068*)^43^. Similarly, marker RM6966 from this study suggested that three QTLs were controlling root traits of low-P tolerance in rice, which were co-localized with the QTLs *qRS8b, qRDW8,* and *qRN8b* reported by Li et al.^19^ for root traits located in the same genomic region under P deficiency with a PV of 11.04%. The R^2^ values of traits associated with marker RM6966 varied from 7.7% to 10.7%. The well-documented high-affinity phosphate transporter (*OsPT6*) gene located on chromosome 8 at 28.3 Mb within this QTL region proved to be involved in long-distance transport of P from root to shoot, and it aids in the accumulation of biomass^44^ under P-deficient conditions. *Insilico* coexpression pattern of the gene *Os08g0564000* revealed that most of the genes involved in root development and signaling pathways during abiotic stress (phosphoesterase family protein; *OsNPC4*)^45^. The coexpression network and expression analysis suggest that the resulting gene network provides the molecular pathways and the biological function of each gene. This piece of information would be useful to explore the role of novel genes related to the target trait of interest in the breeding program.

On the other hand, marker RM242 located on chromosome 9 was a hotspot for biomass accumulation under P-deficient conditions, for which two important QTLs, *qLL 9.1* and *qNT 9.1*, were identified to be associated with low-P conditions, with R^2^ ranging from 8.50% to 9.97%. Marker RM242 was reported to be associated with increasing root length under irrigated conditions and drought stress and was used in a marker-assisted backcross^46^ to improve root length in rice genotype Kalinga III. Goncharova et al.^42^ also reported RM242 to be involved in increasing relative biomass and nitrogen-use efficiency. The locus *Os09t0491740* is located 0.33 Mb away from marker RM242 involved in auxin efflux situated in the plasma membrane. Similarly, markers RM283 and RM297 were also linked with the growth promoter genes brassinosteroid (*Os01t0178500*) and strigolactone (*Os01t0746400*).

Four PAP markers (1 to 4) associated with purple acid phosphatase were developed at ICAR-NRRI to identify genotypes having candidate gene *OsPAP21b*. The gene *OsPAP21b* was reported to be up-regulated under phosphate deprivation^47^ and proved to be a major plant enzyme involved in releasing P from organophosphates that remain unavailable for plants before mineralization^48^. Among the four markers, PAP1 at 2.42 Mb on chromosome 11 exhibited polymorphism across genotypes, which co-localized with tiller number with PV of 7.6% under P deprivation. Among the *Pup1*-specific markers, the R^2^ value of K20-2 registered a maximum of 11.5% for stem thickness and 10.5% for geometric trait top-view area, followed by the *Pup1* core marker K41, which exhibited association with stem thickness of 9%. Another core marker, K29-3, was associated with root dry weight. Therefore, it is believed that *Pup1* is involved in the accumulation of biomass in addition to early rooting in P-deficient conditions. On the other hand, markers RM521 and RM1272 were associated with mycorrhizal colonization % on chromosomes 2 and 4, respectively, with an R^2^ value of 10.3% and 8.3%. The symbiotic association between the rice plant and soil fungi mycorrhiza is well known under P-deficient conditions and fulfills 80% of P^21–23^.

Here, several reliable associations between traits measured under P deprivation and molecular markers were established. Notably, six markers (RM297, RM30, RM6966, RM242, RM283, and PAP1) would be more useful in predicting the identification of genotypes under P-deprived conditions. Typically, an association panel is a diverse set of populations that harbors multiple alleles at any given locus (which requires identifying favorable alleles rather than using the whole genome for hybridization), which are identified phenotypically. Identification and breeding for favorable alleles of the trait of interest from wild genotypes and landraces into modern cultivars will help breeders arrive at a new improved variety with wider adaptability. Therefore, a set of five potential genotypes (IC459373, Chakhao Aumbi, AC100219, AC100062, and Sekri) having a set of favorable alleles would be used appropriately to improve the PUE of rice. Among the selective potential genotypes, IC459373 and AC100219 contain the QTLs *qNT3.1* and *qNT11.1* for tiller numbers, while Dular had only one QTL *qNT11.1*. However, the tiller number between the three genotypes were uniform even they had additional QTLs for the same trait. A more number of QTLs for same trait might have complimentary effect under stress condition for maximizing the root length, which is highly essential for low P condition. This may be observed in the correlation between tiller number and maximum root length (Fig. 2). Therefore, genotypes having multiple QTL for same trait may be explored to utilize them in the breeding program to improve the PUE in rice. As these genotypes (IC459373 and AC100219) having better performance with additional QTLs, they need to be studied further and we are in it. Besides , the identified alleles need to be evaluated to understand the advantage of using them and their effect on different genetic backgrounds.

Identifying suitable donors and improving PUE in rice, potential candidate genes, and markers associated with these traits under low P is a central research area in rice breeding. Therefore, in this study, we demonstrated a selected population panel having significant trait variations. The positive correlation between the traits explained the possibility of improving genotypes for P-deprived soil in a holistic manner. Notably, a negative association was observed between root P content and biomass. The trait total root surface area had become the major contributor for improved P uptake under deficit P than mycorrhizal colonization. The geometric trait of the top-view area exhibited a positive association with maximum root length and root volume, suggesting using a non-destructive approach in screening genotypes under low-P conditions, which is the first report to the authors knowledge as well as based on available scientific documentation. The genotypes were divided into three groups based on distance and model-based analysis, while gene-specific *Pup1* markers classified the panel into two groups. This suggests that traits other than early root growth (*Pup1-*specific markers) in differentiating low-P tolerance must be documented. This unique panel serves to find the association between traits and primers and facilitates the identification of donors with a combination of adaptive traits necessary for low-P conditions. Further, the identified linked markers are highly valued when determining variation for a target trait. The markers RM259, RM297, RM30, RM6966, RM242, RM184, and PAP1 were highly associated with traits responsible for low-P conditions in rice. The multiple traits related to these genome regions are promising resources for improving and understanding PUE and are also more useful as a breeding tool in predicting genotypes under P-deprived conditions.

## Methods

### Plant materials

The population consisted of 155 rice accessions (Table 1S) comprising 41 wild species (21 *Oryza rufipogon,* 19 *O. nivara*, and 1 *O. spontanea*), 37 landraces, and 77 improved varieties originating from eight provinces of India. Seeds were obtained from ICAR-National Rice Research Institute (NRRI), Cuttack, Odisha; the Regional Research & Technology Transfer Station (RRTTS), Coastal zone, Bhubaneswar; and Orissa University of Agriculture and Technology (OUAT), Bhubaneswar. The seed materials were collected from the NRRI gene bank, and appropriate permission was obtained to collect and use them in the current study. Thus, the study complies with local & national regulations.

### Growing conditions and experimental design

#### Phenotyping for phosphorus stress in a cement tank

All the accessions under study were screened in a cement tank for a low-P tolerance facility located at NRRI, Cuttack (20^o^27′09" N, 85^o^55′57" E, 26 masl). The genotypes were direct-seeded in tanks containing low-P soil (< 3 kg/ha; 0–15-cm layer, pH 4.9) with 20 cm X 15 cm between genotypes in three replicates during June 2019. Average day/night temperatures were 33.6/26.0 °C, and relative humidity was 85.9% in bright sunlight. Before sowing, seeds of all the accessions were heat-treated at 50 °C for 45 h in a hot-air oven to break seed dormancy. The soil was irrigated every other day and, 15 days after sowing, the seedlings were thinned, leaving only two seedlings per hill. Chlorophyll content was measured for three plants of each accession on the 44th day from the date of sowing by using a SPAD meter (SPAD-502, Konica Minolta). On the next day (45th day), the plants were uprooted and morphological traits, shoot length (cm), number of tillers, number of leaves, 3rd-leaf length (cm) and width (cm), stem thickness (mm), maximum root length (cm), and root-shoot length ratio were recorded for three plants of each genotype. Subsequently, root traits (total root length (cm), projected root area (cm^2^), root surface area (cm^2^), average root diameter (mm), root volume (cm^3^), and number of root tips) were recorded for each genotype per replication and analyzed by WinRHIZO Pro 2013e (LA 2400, Regent Instruments Inc.). The plant samples were dried in a hot-air oven at 60 °C for 5–6 days and shoot, root, and total dry weight and root-shoot dry weight ratio were recorded in grams. In addition, the plant's top-view area was measured using open-source *Image J* software^49^. Images of each accession were taken using a 12-megapixel Nikon camera at 1.5-m distance, and the pixels were changed into mm^2^ as the top-view area of the plant^49,50^. To quantify total P, the dried plants were powdered around 300 mg shoot and 90 mg root samples following the phospho molybdo vanadate colorimetric method. P concentration in the digest was determined using a Cystronicx UV Spectrophotometer at 420 nm, and total shoot and root P contents were determined on a mg/g dry weight basis. Additionally, to strengthen these data, mycorrhiza colonization was observed.

To study AM fungal root colonization, root samples were collected from all the genotypes, washed thoroughly, cut into small pieces (0.5 to 0.7 cm), and put in falcon tubes containing 15 to 20 ml of 10% KOH. Care was taken when the root samples became immersed inside the KOH solution. Further, the samples were autoclaved at 121 °C at 15 psi pressure for 15 min and washed under running tap water until the roots became clear in color. Each sample was treated with 10 to 15 ml of 2% HCL for 5 min and washed under running tap water. Finally, 10 to 15 ml of 0.05% trypan blue in lactoglycerol was added depending upon root volume and this was left overnight at room temperature. Root samples were rinsed with lactoglycerol (without stain) and slides were prepared with 10 root pieces on each slide for observation under a stereomicroscope to check the root colonization. Percentage of root colonization was determined as per the formula described by McGongigle et al.^51^ : Percentage of colonization = (number of root segments colonized/total number of root segments) × 100.

### Statistical analysis

To study the effect of variation due to P in 155 accessions, descriptive statistics, box plot, ANOVA, and broad-sense heritability (H^2^) were calculated using Windostat 7.5 software with 23 traits measured under low-P conditions. Principal component analysis (PCA) was performed on a matrix of morphometric and geometric traits to assess the variability in traits for tolerance of P deficiency with 155 rice genotypes to differentiate the genotypes based on their performance under low-P conditions. The PCA analysis was executed using the PCA function from the FactoMine R package^52^ in R. The corrplot functions from the corrplot package^53^ in R (version 3.6.3) were used to find the effect of low P on the various genotypes.

### Genotyping

#### DNA isolation, quantification, and PCR amplification

Based on the level of tolerance and phenotypic information, a panel of 120 accessions was shortlisted from 155 accessions for genotypic study based on their distribution pattern. The total genomic DNA was extracted from young leaves following the modified CTAB method^54^. Further, the DNA samples were quantified by a Nanodrop spectrophotometer (Thermo Scientific) and diluted to a working stock of 50 ng µl^−1^. PCR amplified the isolated DNA samples with a 10 μl reaction containing 20 ng of DNA, 10 mM Tris–HCl, 1.5 mM MgCl_2_, 0.2 unit of Taq DNA polymerase (New England Biolabs), 50 μM of dNTPs (New England Biolabs), and 0.1 μM each of forward and reverse primers using a T100 (Bio-Rad, USA) thermal cycler. Thermal cycler reaction was performed as mentioned: initial denaturation at 94 ºC for 10 min, the mixture was cycled 35 times at 94 ºC for 45 s for denaturation, followed by annealing at 55–60 ºC for 45 s (following the TM values of the primer) and 72 ºC of extension for 60 s, and followed by a final extension at 72 ºC for 10 min. The samples were run on a 3.5% agarose gel by using bromophenol blue as a dye and a 50 bp ladder (New England Biolabs) for 1 h in 0.5X tris–acetic acid–EDTA (TAE) buffer. The resolved PCR bands were documented in a gel documentation system (Gel Doc XR + , Bio-Rad, USA), and the images were stored for analysis. In this study, with the prior information, 78 low-P QTL-linked primers (Table 2), including 13 *Pup1*-specific markers^55^, were used to map the QTLs associated with traits related to low P and the genetic architecture of the population panel.

#### Genetic diversity, population structure, and association analysis

The molecular size of the amplified fragments was determined by image lab software using 50 bp DNA ladders as the standard. Amplification of DNA samples with the primers was scored as per the molecular size or '1' for amplified regions and '0' for unamplified regions. A data matrix with '0' and '1' or the molecular size was prepared depending on the amplification, and the data matrix underwent further analysis. Genetic diversity parameters, major allele frequency, gene diversity, heterozygosity, and the polymorphic information content (PIC) were calculated for each SSR locus using Power marker software version 3.25^56^. An un-rooted tree with a bootstrap value of 1000 was used to construct an unweighted neighbor-joining tree (Nei 1972) using Darwin 6.0 software^57^. Analysis of molecular variance (AMOVA) was generated using the GenAlex 6.502 program to describe the presence of molecular variance components within and between the population differentiation among the five assumed sub-populations^58^ and to estimate the F statistics comprising the deviations from Hardy–Weinberg expectation across the population (F_IT_), within-population (F_IS_), and for correlation of alleles between the sub-populations (F_ST_). In addition, Shannon's Information index observed and expected homozygosity, observed and expected heterozygosity, Nei's genetic diversity index, and the number of migrants (NM) between the assumed sub-populations were calculated by POPGENE program version 1.31^59^.

The genotypic data of the genotypes under study were analyzed for possible population structure with the model-based program STRUCTURE 2.3.4^60^ using a length of the burn-in period of 1,00,000, followed by 10,000 Markov chain Monte Carlo (MCMC) replications. At least ten runs of STRUCTURE were performed by setting the number of sub-populations (K) from K = 1 to K = 10. To find the true K-value, ad hoc statistics ∆K was followed^61^ using Structure Harvester version 0.6.94^62^. In this study, both model and distance-based cluster analysis were carried out with the data generated with all 78 primers collectively, excluding 13 Pup1-specific markers and only with Pup1-specific markers to realize the distribution of the *PSTOL1* gene across species and the significance of other markers under low P. Further, to determine the genetic relatedness between traits measured under low P, the rice genotypes and primers were analyzed by the general linear model (GLM) and mixed linear model (MLM) in TASSEL version 5.2.63^63^. False discovery rate (FDR) was used to obtain *q-*values (adjusted P-values) as described by earlier studies^64^. A significant association between markers and traits was identified based on their R^2^ and P-value.

#### Candidate gene analysis underlying a QTL region and coexpression of gene assay

The associated traits and SSR markers aligned to the IRGSP 1.0 genome in The Rice Annotation Project (https://rapdb.dna.affrc.go.jp/viewer/gbrowse/irgsp1/) to retrieve the genes associated with linked markers. The sequence on either side of each linked marker extended from 500 kb left and 500 kb right and was marked as a QTL region and meticulously searched to find the genes associated with low P tolerance. The interval on either side of the associated marker can be called an interval of the QTL genome^65^. Furthermore, we have performed the coexpression of gene assay for each putative candidate gene based on the guide gene approach using the riceFREND database (https://ricefrend.dna.affrc.go.jp/) to identify the coexpressed genes. This approach provided the details of coexpressed genes, functional information associated with reported genes, and transcription factors. The direct link of this database with RiceXPro (https://ricexpro.dna.affrc.go.jp/) helps to understand the insilico expression of these candidate genes. This integrative process in two databases allows the number of nodes of the gene interactions, and the ranking of nodes helps identify the groups of functionally related genes^66^.

### Ethics approval and consent to participate

The authors declare that this research review was conducted in the absence of any commercial or economic associations that could be construed as potential conflicts of interest.

## Supplementary Information

Supplementary Figure S1.

Supplementary Table S1.

Supplementary Table S2.

## Data Availability

The comprehensive collected data and information supporting the conclusions of this research article are provided as figures, tables, and supplementary tables.
