# Supplementary material for: Multi-trait association study identifies loci associated with tolerance of low phosphorus in Oryza sativa and its wild relatives
Source: Sci Rep. 2022 Mar 8;12:4089. doi: 10.1038/s41598-022-07781-5 (PMC8904515; doi:10.1038/s41598-022-07781-5)
Supplement: Supplementary file 1 — Supplementary Figure S1. [file 41598_2022_7781_MOESM1_ESM.pptx]

## Slide 1
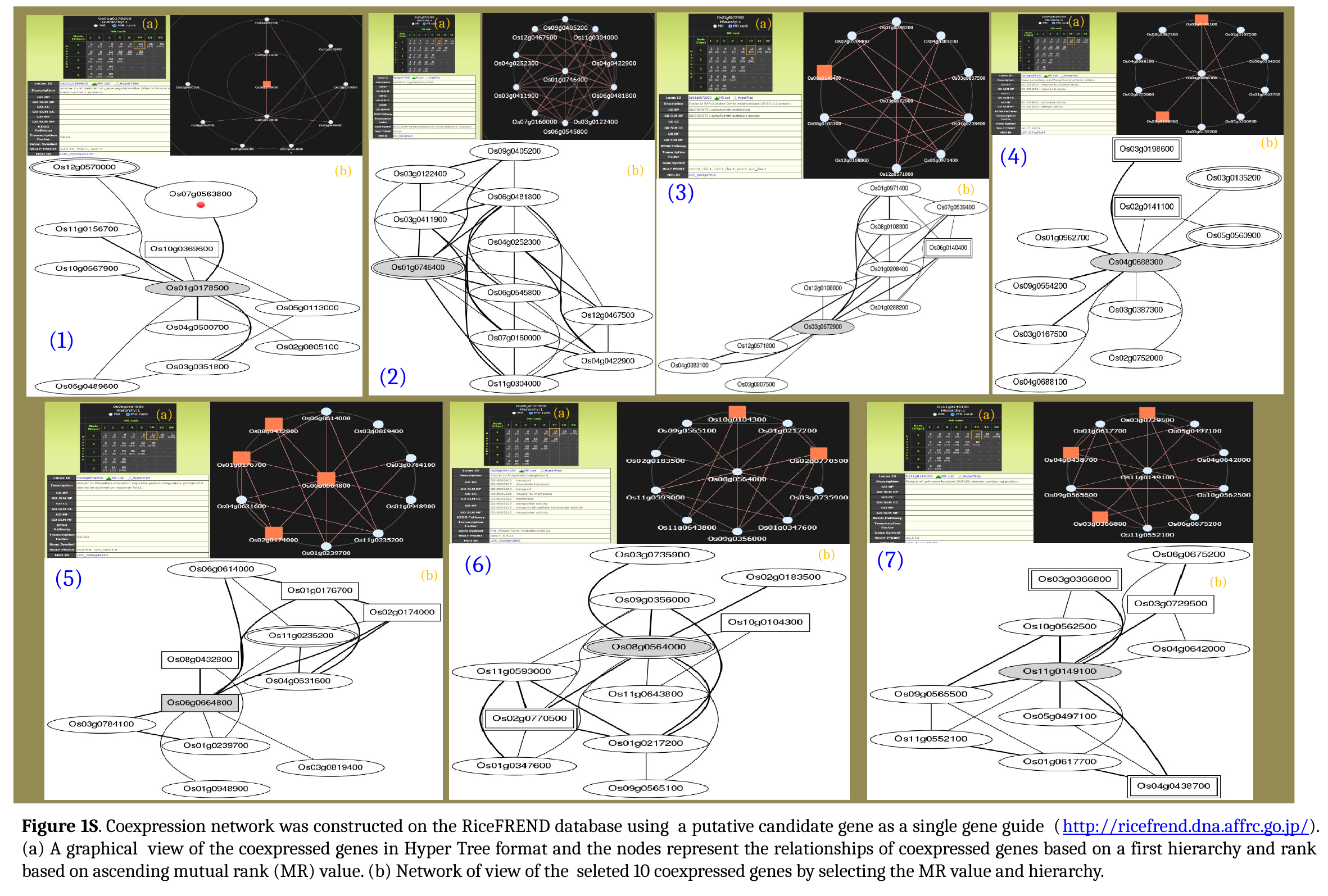

(a)
(a)
(a)
(a)
(4)
(3)
(1)
(2)
(7)
(6)
(5)
(b)
(b)
(b)
(b)
(a)
(a)
(a)
(b)
(b)
(b)
Figure 1S. Coexpression network was constructed on the RiceFREND database using a putative candidate gene as a single gene guide (http://ricefrend.dna.affrc.go.jp/). (a) A graphical view of the coexpressed genes in Hyper Tree format and the nodes represent the relationships of coexpressed genes based on a first hierarchy and rank based on ascending mutual rank (MR) value. (b) Network of view of the seleted 10 coexpressed genes by selecting the MR value and hierarchy.
