# Supplementary material for: Multi-trait association study identifies loci associated with tolerance of low phosphorus in Oryza sativa and its wild relatives
Source: Sci Rep. 2022 Mar 8;12:4089. doi: 10.1038/s41598-022-07781-5 (PMC8904515; doi:10.1038/s41598-022-07781-5)
Supplement: Supplementary file 2 — Supplementary Table S1. [file 41598_2022_7781_MOESM2_ESM.docx]

**Table 1S** List of accessions of different Oryza sp. used for evaluating in P deprive soil

| **S.No.** | **Genotype name/Accession number** | **Species/Landrace**  **/Improved** | **Ecology** | **Place of collection (Province)** | **S.No.** | **Genotype name/Accession number** | **Species/Landrace**  **/Improved** | **Ecology** | **Place of collection (Province)** |
| --- | --- | --- | --- | --- | --- | --- | --- | --- | --- |
| 1 | Dular | Landrace | Upland | Odisha | 25 | AC100301 | *O. nivara* | Wild | Odisha |
| 2 | Kasalath | Landrace | Upland | Assam | 26 | AC100295 | *O. spontanea* | Wild | Odisha |
| 3 | IC459373 | Landrace | Upland | Assam | 27 | AC100296 | *O. nivara* | Wild | Odisha |
| 4 | CR Dhan 801 | Improved variety | Rainfed lowland | Odisha | 28 | AC100285 | *O. nivara* | Wild | Odisha |
| 5 | AC100281 | *O. rufipogon* | Wild | Odisha | 29 | AC100203 | *O. nivara* | Wild | Odisha |
| 6 | AC100062 | *O. rufipogon* | Wild | Odisha | 30 | AC100219 | *O. rufipogon* | Wild | Odisha |
| 7 | AC100326 | *O. rufipogon* | Wild | Odisha | 31 | Kabuk Phou | Landrace | Upland | Manipur |
| 8 | AC100035 | *O. rufipogon* | Wild | Odisha | 32 | Chakhao Aubi | Landrace | Upland | Manipur |
| 9 | AC100142 | *O. nivara* | Wild | Odisha | 33 | Kumbhi Phou | Landrace | Upland | Manipur |
| 10 | AC100021 | *O. nivara* | Wild | Odisha | 34 | Akhiyaturfa | Landrace | Upland | Manipur |
| 11 | AC100032 | *O. nivara* | Wild | Odisha | 35 | Leimaphou | Landrace | Irrigated | Manipur |
| 12 | AC100284 | *O. rufipogon* | Wild | Odisha | 36 | Longmanabi | Landrace | Upland | Manipur |
| 13 | AC100329 | *O. nivara* | Wild | Odisha | 37 | CR Dhan 103 | Improved variety | Upland | Odisha |
| 14 | AC100193 | *O. rufipogon* | Wild | Odisha | 38 | Khitish | Improved variety | Irrigated | Odisha |
| 15 | AC100042 | *O. rufipogon* | Wild | Odisha | 39 | Sadabahar | Improved variety | Upland | Odisha |
| 16 | AC100015 | *O. rufipogon* | Wild | Odisha | 40 | Neela | Improved variety | Upland | Odisha |
| 17 | AC100282 | *O. rufipogon* | Wild | Odisha | 41 | Abhishek | Improved variety | Irrigated | Odisha |
| 18 | AC100010 | *O. nivara* | Wild | Odisha | 42 | Harishankar | Landrace | Upland | Odisha |
| 19 | AC100006 | *O. rufipogon* | Wild | Odisha | 43 | Kamesh | Improved variety | Upland | Odisha |
| 20 | AC100293 | *O. nivara* | Wild | Odisha | 44 | Phalguni | Improved variety | Upland | Odisha |
| 21 | AC100189 | *O. rufipogon* | Wild | Odisha | 45 | Kowni | Landrace | Irrigated | Tamil Nadu |
| 22 | AC100328 | *O. nivara* | Wild | Odisha | 46 | AC100175 | *O. nivara* | Wild | West Bengal |
| 23 | AC100309 | *O. rufipogon* | Wild | Odisha | 47 | AC100142(A) | *O. nivara* | Wild | Odisha |
| 24 | AC100107 | *O. nivara* | Wild | Odisha | 48 | ASD16 | Improved variety | Irrigated | Tamil Nadu |
| **S.No.** | **Genotype name/Accession number** | **Species/Landrace**  **/Improved** | **Ecology** | **Place of collection (Province)** | **S.No.** | **Genotype name/Accession number** | **Species/Landrace**  **/Improved** | **Ecology** | **Place of collection (Province)** |
| 49 | Poongar | Landrace | Irrigated | Tamil Nadu | 71 | Uphar | Improved variety | Rainfed lowland | Odisha |
| 50 | AC100135 | *O. rufipogon* | Wild | Odisha | 72 | Ramchandi | Improved variety | Rainfed lowland | Odisha |
| 51 | AC100117 | *O. nivara* | Wild | Odisha | 73 | Sebati | Improved variety | Irrigated | Odisha |
| 52 | Sekri | Landrace | Upland | Uttar Pradesh | 74 | Vandana | Improved variety | Upland | Jharkhand |
| 53 | Longmanabi(A) | Landrace | Upland | Manipur | 75 | Sneha | Improved variety | Upland | Odisha |
| 54 | Sukhapnki | Landrace | Upland | Manipur | 76 | Ramba | Improved variety | Rainfed lowland | Odisha |
| 55 | Lalat MAS | Improved variety | Irrigated | Odisha | 77 | Pooja | Improved variety | Lowland | Odisha |
| 56 | Meher | Improved variety | Irrigated | Odisha | 78 | Mahanadi | Improved variety | Rainfed lowland | Odisha |
| 57 | Rajeswari | Improved variety | Irrigated (coastal saline) | Odisha | 79 | Annapurna | Improved variety | Upland | Odisha |
| 58 | Subhadra | Improved variety | Upland | Odisha | 80 | Vanaprabha | Improved variety | Upland | Odisha |
| 59 | Asutosh | Improved variety | Irrigated | Odisha | 81 | Jagannath | Improved variety | Rainfed lowland | Odisha |
| 60 | Hiranmayee | Improved variety | Irrigated | Odisha | 82 | Udayagiri | Improved variety | Upland | Odisha |
| 61 | Pratap | Improved variety | Irrigated | Odisha | 83 | Samanta | Improved variety | Irrigated | Odisha |
| 62 | Lalitagiri | Improved variety | Upland | Odisha | 84 | Birupa | Improved variety | Irrigated | Odisha |
| 63 | Mrunalini | Improved variety | Rainfed lowland | Odisha | 85 | Rudra | Improved variety | Upland | Odisha |
| 64 | Pradeep | Improved variety | Irrigated | Odisha | 86 | Ghanteswari | Improved variety | Upland | Odisha |
| 65 | Pathara | Improved variety | Upland | Odisha | 87 | Bhubana | Improved variety | Irrigated | Odisha |
| 66 | Pratibha | Improved variety | Irrigated | Odisha | 88 | Tanmayee | Improved variety | Irrigated | Odisha |
| 67 | Ranidhan | Improved variety | Rainfed lowland | Odisha | 89 | Sabitree | Improved variety | Rainfed lowland | Odisha |
| 68 | Suphala | Improved variety | Upland | Odisha | 90 | Daya | Improved variety | Irrigated | Odisha |
| 69 | Sarathi | Improved variety | Irrigated | Odisha | 91 | Bhoi | Improved variety | Irrigated | Odisha |
| 70 | Manaswini | Improved variety | Irrigated | Odisha | 92 | Parijat | Improved variety | Upland | Odisha |

| **S.No.** | **Genotype name/Accession number** | **Species/Landrace**  **/Improved** | **Ecology** | **Place of collection (Province)** | **S.No.** | **Genotype name/Accession number** | **Species/Landrace**  **/Improved** | **Ecology** | **Place of collection (Province)** |
| --- | --- | --- | --- | --- | --- | --- | --- | --- | --- |
| 93 | Kalinga 3 | Improved variety | Upland | Jharkhand | 116 | Heera | Improved variety | Upland | Odisha |
| 94 | Indarbati | Improved variety | Rainfed lowland | Odisha | 117 | Khandagiri | Improved variety | Upland | Odisha |
| 95 | Sidhanta | Improved variety | Upland | Odisha | 118 | Hema | Improved variety | Irrigated | Odisha |
| 96 | Urbasi | Improved variety | Rainfed lowland | Odisha | 119 | Manika | Improved variety | Rainfed lowland | Odisha |
| 97 | Jagabandhu | Improved variety | Rainfed lowland | Odisha | 120 | Annada | Improved variety | Upland | Odisha |
| 98 | Jajati | Improved variety | Irrigated | Odisha | 121 | CR Dhan 40 | Improved variety | Upland | Odisha |
| 99 | Kanchan | Improved variety | Rainfed lowland | Odisha | 122 | IR36 | Improved variety | Irrigated | IRRI |
| 100 | Tejaswini | Improved variety | Irrigated | Odisha | 123 | A. kuruvai | Landrace | Irrigated | Tamil Nadu |
| 101 | Hasanta | Improved variety | Rainfed lowland | Odisha | 124 | A. kuruvai(A) | Landrace | Irrigated | Tamil Nadu |
| 102 | Surendra | Improved variety | Irrigated | Odisha | 125 | H24 | Landrace | Irrigated | West Bengal |
| 103 | Badami | Improved variety | Upland | Odisha | 126 | Black puttu | Landrace | Irrigated | Tamil Nadu |
| 104 | Prachi | Improved variety | Rainfed lowland | Odisha | 127 | Brown gora | Landrace | Upland | Bihar |
| 105 | Gajapati | Improved variety | Irrigated | Odisha | 128 | Burma black | Landrace | Irrigated | West Bengal |
| 106 | Pratikshya | Improved variety | Irrigated | Odisha | 129 | Chakhao | Landrace | Upland | Manipur |
| 107 | Bhanja | Improved variety | Irrigated | Odisha | 130 | Chakhao(A) | Landrace | Upland | Manipur |
| 108 | Kharavela | Improved variety | Irrigated | Odisha | 131 | Chakhao Aubi(A) | Landrace | Upland | Manipur |
| 109 | Shankar | Improved variety | Upland | Odisha | 132 | Chakhao Poreition | Landrace | Upland | Manipur |
| 110 | Keshari | Improved variety | Upland | Odisha | 133 | IC418443 | Landrace | Upland | Andaman & Nicobar |
| 111 | Anjali | Improved variety | Upland | Odisha | 134 | IC450521 | Landrace | Upland | Odisha |
| 112 | Gouri | Improved variety | Irrigated | Odisha | 135 | IC544887 | Landrace | Upland | Burma |
| 113 | Konark | Improved variety | Irrigated | Odisha | 136 | Kabuk phou(A) | Landrace | Upland | Manipur |
| 114 | Nilagiri | Improved variety | Upland | Odisha | 137 | Kaliabhat | Landrace | Irrigated | West Bengal |
| 115 | Mahalaxmi | Improved variety | Rainfed lowland | Odisha | 138 | Kaliabhat(A) | Landrace | Irrigated | West Bengal |

| **S.No.** | **Genotype name/Accession number** | **Species/Landrace**  **/Improved** | **Ecology** | **Place of collection (Province)** |
| --- | --- | --- | --- | --- |
| 139 | Manipuri black | Landrace | Upland | Manipur |
| 140 | Manipuri black(A) | Landrace | Upland | Manipur |
| 141 | Mummy hunger | Landrace | Upland | Manipur |
| 142 | Baman Phou | Landrace | Upland | Manipur |
| 143 | Bulu harana | Landrace | Upland | Manipur |
| 144 | Gini | Landrace | Upland | Manipur |
| 145 | AC100062(A) | *O. rufipogon* | Wild | Odisha |
| 146 | AC100133 | *O. rufipogon* | Wild | Odisha |
| 147 | AC100169 | *O. rufipogon* | Wild | West Bengal |
| 148 | AC100170 | *O. rufipogon* | Wild | West Bengal |
| 149 | AC100209 | *O. rufipogon* | Wild | Odisha |
| 150 | AC100219(A) | *O. rufipogon* | Wild | Odisha |
| 151 | AC100281(A) | *O. rufipogon* | Wild | Odisha |
| 152 | AC100032(A) | *O. nivara* | Wild | Odisha |
| 153 | AC100121 | *O. nivara* | Wild | Odisha |
| 154 | AC100123 | *O. nivara* | Wild | Odisha |
| 155 | AC100283 | *O. nivara* | Wild | Odisha |

S.No. 1-120 used for genotypic studies
